# Special Features of Polyester-Based Materials for Medical Applications

**DOI:** 10.3390/polym14050951

**Published:** 2022-02-27

**Authors:** Raluca Nicoleta Darie-Niță, Maria Râpă, Stanisław Frąckowiak

**Affiliations:** 1 Physical Chemistry of Polymers Department, Petru Poni Institute of Macromolecular Chemistry, 41A Grigore Ghica Voda Alley, 700487 Iasi, Romania; darier@icmpp.ro; 2 Faculty of Materials Science and Engineering, University Politehnica of Bucharest, 313 Splaiul Independentei, 060042 Bucharest, Romania; 3 Faculty of Environmental Engineering, University of Science and Technology, 50-013 Wrocław, Poland; stanislaw.frackowiak@pwr.edu.pl

**Keywords:** polyesters, medical applications, biomaterial, processing methods, properties, COVID-19, risks

## Abstract

This article presents current possibilities of using polyester-based materials in hard and soft tissue engineering, wound dressings, surgical implants, vascular reconstructive surgery, ophthalmology, and other medical applications. The review summarizes the recent literature on the key features of processing methods and potential suitable combinations of polyester-based materials with improved physicochemical and biological properties that meet the specific requirements for selected medical fields. The polyester materials used in multiresistant infection prevention, including during the COVID-19 pandemic, as well as aspects covering environmental concerns, current risks and limitations, and potential future directions are also addressed. Depending on the different features of polyester types, as well as their specific medical applications, it can be generally estimated that 25–50% polyesters are used in the medical field, while an increase of at least 20% has been achieved since the COVID-19 pandemic started. The remaining percentage is provided by other types of natural or synthetic polymers; i.e., 25% polyolefins in personal protection equipment (PPE).

## 1. Introduction

In addition to other types of polymeric materials, polyesters have found diverse uses in biomedical applications, such as controlled drug release systems [1,2,3,4,5], time-tailored implants, screws, prostheses, and different 3D structures including scaffolds for bone reconstruction and tissue engineering [6]. Various medical products containing polyesters are commercially available, while new ones are awaiting patents for placement on the market.

Polyesters such as poly(lactic acid) (PLA), poly-L-lactide (PLLA), poly(ε-caprolactone) (PCL), poly(glycolic acid) (PGA), poly(lactic-glycolic acid) (PLGA) copolymers, or poly(hydroxyalkanoates) (PHA) are synthetic biodegradable polymers highly used in medical applications due to their wide range of custom properties, availability, tailoring capacity, cost-effectiveness, and easy processing. Since its development in 1932 by DuPont and the establishment of the first large production facility by Cargill Dow Polymers in 2001, PLA has experienced rapid growth, with a high potential to replace conventional petrochemical-based polymers in many medical applications. Before being produced on a larger scale, PLA was mainly used in medical applications due to its relatively high cost. Although most polyesters are synthesized from carbohydrate petroleum-based sources, alternative sustainable raw materials were found, with PLA, poly(hydroxybutyrate) (PHB), and partially bio-based polyethylene terephthalate (PET) being derived from renewable sources.

The polar characteristics of a polymer are among the most important properties to be considered in medical applications such as cell regeneration and tissue engineering, as variations in hydrophobicity lead to different interactions of scaffolds with cells and proteins (targeting cell attachment, spread, and viability in biological systems) [7]. From the medical point of view, the most important ones are inert nature and biocompatibility.

Polyester materials are widely studied for the development of biological tissue that can enable the restoration and maintenance of the functions of damaged human organs or tissues. This is due to the fact that esters, of which polyester materials are composed, exist naturally in the human body; i.e., fatty acids are energy sources and membrane constituents. They have biological activities that act to influence cell and tissue metabolism, function, and responsiveness to hormonal and other signals [8].

Tissue engineering can be considered an alternative to conventional more invasive surgical procedures when it comes to replacing or restoring damaged organ or tissue. The global market for tissue engineering was estimated at USD 9.9 billion in 2019, and is expected to register a compound annual growth rate (CAGR) of about 14.2% between 2020 and 2027 [9]. The main types of tissue engineering are cells, tissue-inducing substances, and scaffolds, which are basically cells combined with a type of matrix that can provide a physical support and allow the tissue growth. Adequate mechanical stiffness is required for polyesters such as PCL and PLA intended to be used as body tissues in order to prevent new-tissue deformation and overcome in vivo stresses [6,10].

The more conventional approaches are divided mainly into autografting and allografting. In order to introduce respective treatments, tissue is transplanted within the patient from one site to another or between two different patients. Both approaches have their drawbacks; i.e., anatomical restrictions, the risk of transferring diseases between the patients, and a possible rejection response from the patient’s immune system [11]. 

Polyesters are naturally biodegradable materials due to the fact that the ester bonds can be broken down by the means of hydrolysis or esterases, and in some cases, the degradation process can be undertaken by both of the factors. The hydrolytic degradation is one of the key features behind why these materials are of growing popularity when it comes to tissue-engineering studies, as they can be engineered to yield nontoxic products that are metabolized by the human body [12]. The ability to degrade in vivo is crucial for tissue-engineering applications, as there is need for a smooth and certain transition of functionality from the degrading polymeric scaffold to newly grown tissue. As time is very important in this process, it is possible to tailor the rate of the degradation by changing the chemical structure of the polymer or its additives [13]. 

There are two different mechanisms for polyester degradation that can affect the implementation of certain polymers: surface and bulk erosion. In surface erosion, the polymer maintains its bulk integrity, as the erosion is limited to the surface of the material. The device will reduce in its dimensions—the walls will become thinner; however, the core and its properties will remain intact. It is worth mentioning that as the degradation process is highly focused on the surface of the immersed material, the mass loss and dimensional stability is strictly proportional to the area of surface that is exposed to water. The other degradation mechanism, bulk erosion, occurs when the rate at which the water penetrates is much greater than the rate at which the polymer is being converted into water-soluble materials. The dimensions of the device may remain unaffected or even will increase with the volumetric water uptake; however, it will result in erosion throughout the material volume. This is a two-step process, as the molecular weight of the material is affected by its gradual decrease, as the properties of the material will tend to downgrade at a certain pace. After exceeding a critical value of the molecular weight with water penetrating, accompanied by the cleaving of the polymer chains, especially the hydrolytically unstable chemical bonds converting longer chains into water-soluble fragments, an enzyme-based attack occurs. Final mass loss is rapid, with a sudden release of degradation products, and then the material disintegrates completely [14]. 

In the case of surgical implant applications, polyesters are in the first generation of commercially available implants, therefore not many scientists have published new polyester blends and composites for such applications since 2016. Most of the literature available on the subject refers to clinical cases that compare those commercial products in a group of patients. 

Wound-dressing materials should have important requirements related to their biocompatibility [15], wound healing [16], wound adhesion [17,18], maintenance of wound moisture [19,20], inhibition of the growth of bacteria [15,21,22], removal of excess exudates, and reductions in the dressing frequency [23,24]. 

Multiresistant infections, especially during the recent COVID-19 pandemic, have affected all of humanity from a variety of perspectives, including health issues, the socioeconomic crisis, and environmental concerns. Despite the economic shock that has affected many industries, the demand for polyester materials has shown great resilience. The use of PLA or PET for the manufacture of personal protective equipment (PPE) has received great consideration [25,26,27], with the polyester market being relieved of its worst consequences. The active integration of nanostructures into polyesters that self-sterilize against pathogens may provide a way to lower the transmission of viral infections. Given the recent growth in various infectious threats, the development of effective vaccination technologies containing novel vaccine delivery vehicles based on polyesters to immunize against various strains of viruses is in high demand. Sanitization is also highly necessary to prevent infection. 

**Figure 1:**
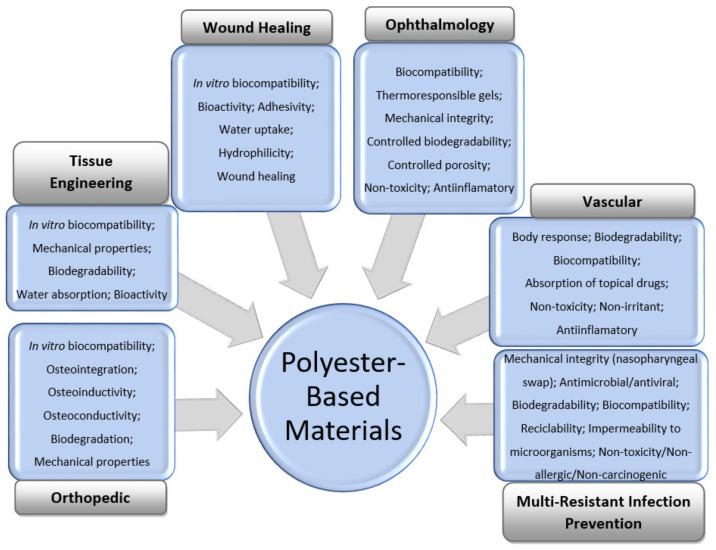
General requirements for the design of polyester-based materials for medical applications.

The general features of polyester-based materials used for orthopedic, tissue-engineering, wound-healing, vascular, and ophthalmology applications, as well as prevention of multiresistant infections, including during the COVID-19 pandemic, are shown in Figure 1.

Neat polyesters can be combined with natural or synthetic materials to increase their bioactivities and obtain the desired properties for each medical application. The main recently designed formulations or composites containing polyesters, their manufacturing methods, and special features for the above-mentioned applications are summarized in this review.

## 2. Orthopedic Applications

Bone defects include trauma, bone infection, osteonecrosis, osteoporosis, bone tumors, and iatrogenic injury. Bone illnesses are expected to increase in the future due to population growth and aging. Therefore, there is a huge need for clear approaches that lead to bone healing. Bone treatment management involves autologous bone grafting, allogeneic grafting, xenografting from other species, or artificial bone-substitute materials [28,29]. Each option has advantages and disadvantages. Among all of them, autologous bone grafting provides an excellent healing capability, but is limited by the quantity of the donor site. Artificial bone-substitute materials represent an alternative to autologous and allogeneic bones, which are traditional options for patients to treat bone defects [28]. Researchers have developed innovative materials that are able to support the full repair of damaged bones. 

The ideal bone-substitute material should be biodegradable in order to eliminate the need for a secondary surgery [30,31] and osteoconductive to promote bone regeneration [32,33,34]. The three-dimensional (3D) scaffold structure should have pore sizes larger than 100 µm and a highly interconnected pore structure to facilitate bone ingrowth, nutrient transport, and degradation of products in an acid–base balance. Polyester materials also should meet the rheological property requirements for printing; namely, the loss modulus (G″) should be greater than the storage modulus (G′) at the printing temperature (Tp), and the melt viscosity should be below 106 mPa·s to permit flow under applied pressure. The local microenvironment may influence the cell growth and bone repair, so it should be maintained at pH 7.2–7.4 [35]. Since the acidic degradation product of pure polyesters restrains the growth of cells or tissues, it is necessary to find new strategies for neutralization of the acidic condition that results from degradation of products. Finally, polyester biomaterials often require bioactivity to control cell function, including cell migration (infiltration), proliferation, and phenotype preservation or differentiation.

Usually, materials used to repair bone defects are metallic biomaterials [36,37], bioceramics [38,39], and natural and synthetic polymers [40,41]. Hydroxyapatite (HA; (Ca_10_(PO_4_)_6_(OH)_2_)) has been used as filler in polymer composites to improve the biocompatibility, mechanical strength, and porosity of biomaterials due to its similarity in structure and composition to bone and enamel, or to create polyester nanografts that impart the biodegradability and bioresorbability of polymers with their osteoconductivity, osteoinductivity, and osteointegration properties [42,43]. 

Naturally occurring polymers display inherent bioactivity, which is not the case for synthetic polymers [44]. Therefore, it is of great interest to design thermoplastic polyesters, such as PLA, PCL, PHA and thermoplastic polyurethane (TPU), for use as matrices in a wide range of bone applications. 

### 2.1. Neat Polyesters 

PLA is commercially used for pins for the foot, ankle, knee, and shoulder. PLLA and PDLA are two stereoisomers of PLA currently used in bone applications. PLLA is used for screws, washers, pins, rods, and plates used in cranial, oral, maxillofacial, plastic, and reconstructive surgeries, while blends of PLLA with HA, PLG, and PGA are employed for orthopedic fracture fixation devices [45]. Although the PLA absorbable reinforcement ligaments showed slow enzymatic degradation rates, they recorded high values as compared with the PLA synthesized in the laboratory, and were found entirely biocompatible according to the in vivo and in vitro hydrolysis in the human body [46,47]. While PLLA is degraded during 2–5 years in phosphate-buffered saline (PBS) (pH 7.4) at 37 °C, 2 months are needed for poly(D,L-lactic acid) (PDLLA) to lose its integrity, and 1 year under similar conditions for complete degradation. Currently, PLA composites are used in small load-bearing applications.

PCL has been widely used in the fabrication of 3D scaffolds in the field of bone-tissue engineering due to its advantages such as good biocompatibility, a slow degradation rate, released products that are less acidic in comparison to other polyesters, and its potential in load-bearing applications. 

The chemical surface of a polyester-based material designed for orthopedic applications can be modified by associating with hydrophilic and hydrophobic polymers or mixing it with HA or halloysite nanotubes (HNTs), resulting in cell adhesion or enhancement of the mechanical properties. For example, Torres et al. [48] combined hydrophobic PCL with PLA and hydrophilic poly(2-hydroxyethyl methacrylate) (PHEMA) with ethyl methacrylate (EMA) and evaluated the effects of chemical surface modifications on cell viability, proliferation, and morphology. Higher cell viability was recorded for moderately hydrophobic surfaces within 3 days, while more hydrophilic surfaces reached advanced cell proliferation at prolonged culture periods. For moderated hydrophobic PCL/PLA material, the scanning electron microscopy (SEM) results showed round-shaped cells or cluster formations, with a monolayer of cells partially adhered to the polymeric surface, and improved cell viability with addition of HA and HNTs. Variations in wettability influenced the protein absorption on the surface of the biomaterial; for example, albumin easily adhered to the polyester surface due to the hydrophobic affinity.

PHAs are next-generation biomaterials isolated from bacterial sources designed for development on bone marrow cells and scaffolds for bone-tissue applications [33,49]. PHAs are highly biocompatible natural polyesters that degrade into water, carbon dioxide, and D-3-hydroxybutyric acid, a common metabolite that occurs in living organisms, avoiding the occurrence of inflammatory reactions developed in the case of other synthetic polyesters. PHB and poly(hydroxybutyrate-co-valerate) (PHBV) are the main homopolymer representants of the PHA family. PHB shows high mechanical strength, biocompatibility, and easy processability. The low narrow thermal processing window, lack of toughness, hydrophilicity, and bioactivity are the main drawbacks of PHA [50]. 

The long-term degradation of thermoplastic polyurethane (TPU) filaments in phosphate-buffered saline (PBS) and cell proliferation indicated that the polyurethanes (PUs) also are attractive polyester materials for bone-tissue applications [51]. 

Although pure polyesters are biodegradable and bioresorbable, without modifications they do not possess enough rigidity for resistance during implant insertion, and the degradative products can cause inflammatory reactions, so they cannot be used for orthopedic applications. These limitations of thermoplastic polyesters can be overcome by introducing bioactive ceramics such as HA, tricalcium phosphate (TCP), and bioactive glass (BG) [52,53,54]. The literature revealed that polyesters mixed with HA [55,56], PLA, and PGA mixed with BG [57,58] have been extensively studied as materials for orthopedic applications in terms of their processing, physicochemical, mechanical, and in vitro biological properties. Mixtures of polyesters with HA used for artificial bone substitute materials can show excessive hardness and brittleness, as well as the occurrence of foreign bodies, which cause an acidic microenvironment, and are dangerous to cell proliferation and bone regeneration. A strategy to prevent an acidic environment and an aseptic inflammation reaction was given by Kuo et al. [59]. Accordingly, the porous biodegradable structure based on β-TCP and poly(l-lactic-co-glycolic acid) (PLGA) was degraded 37% into smaller molecules during 60 days of accelerated testing [59]. The reaction during degradation was an acid–base neutralization due to the alkaline environment provided by the degradation product of TCP. 

Even if PCL is incompatible with bioceramic materials, PCL/nano-HA electrospun fibers with improved mechanical characteristics were achieved by using a compatibilizer, such as PCL/poly(ethylene phosphoric acid) (PEPA) block-copolymer [60]. These nanocomposites showed that vancomycin was released against *Staphylococcus aureus (S. aureus).*

### 2.2. Manufacturing of Polyesters with Improved Functionalities

Different methods for fabrication of polyester-based materials for orthopedic applications are presented in Table 1. 

The usual techniques for preparing modified polyesters for artificial bone-substitute materials involve 3D printing [51,61,65,66,72,74,75,76], thermally induced phase separation (TIPS) [32], salt leaching [32,69,79], solvent casting [33,35,77,79], electrospinning [60,64,70], copolymerization [31], and polycondensation [73]. 

The addition of silica aerogel to PCL has been reported to lead to biomaterial scaffolds obtained by the solvent-casting method with a stabilized environmental pH, enhanced cell viability, and osteogenic activity [35].

Macroporous PHBV films with hydroxyvalerate (HV) varying from 0 to 12 wt % were prepared by solvent casting using NaCl as a porogen for investigation of the effect of HV on the degradation of films and osteoblastic cell growth [33]. It was demonstrated that the PHBV film with a content of 12 wt % HV content could be used for orthopedic applications. The solvent-casting method was also used to obtain a potential bone scaffold material by mixing PCL with silica (SiO_2_) aerogels [35]. At a weight ratio of 1:0.5 between PCL and SiO_2_, the composite had a constant pH environment for up to 4 weeks, providing better NIH3T3 cell survival.

Scaffolds based on plasticized PHB and bacterial cellulose (BC) up to 2 wt % were prepared by melt-mixing and salt-leaching techniques and used for healing critical-size calvaria defects [69]. It was demonstrated that the smallest intraosseous defect was filled with new mature bone at 20 weeks postimplantation, due to the breaking of beta (1–4) glycosidic linkages in BC, which supported a progressive mineralization of scaffolds.

**Figure 2:**
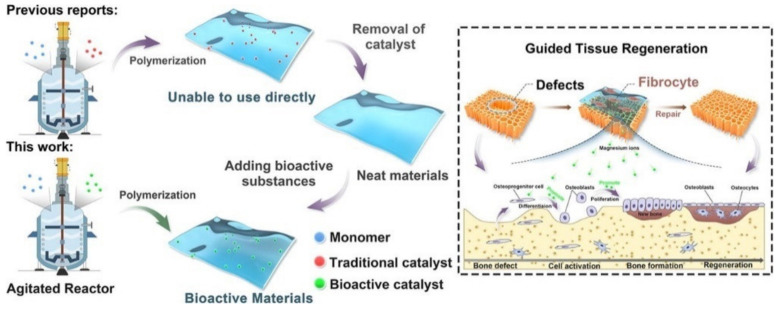
In-reactor engineering of bioactive aliphatic polyesters. Reproduced from [31] with permission from Elsevier.

The copolymerization of poly(butylene succinate) (PBSu) with sebacic acid (SeA) in the presence of a magnesium catalyst formed in situ at a large scale is a new approach for the direct in-reactor engineering of bioactive polyesters without the use of a toxic catalyst [31] (Figure 2). First, PBSu was synthesized by two-stage esterification of succinic acid (SA) and 1,4-butanediol (BDO). It was proved that the low-cost bioactive polyester could be used to guide tissue regeneration, due to the higher degree of bone formation rate after 16 weeks as compared with a commercial PLA membrane. These features, together with cell proliferation, osteogenic activity, and anti-inflammatory properties of the PBSu/SeA composition, were assigned to magnesium ions.

Another paper reported a polyester/HA graft synthesized by “graft-from” polymerization of D,L-lactide with HA in the presence of a tin(II)-2-ethylhexanoate catalyst, which was used as additive for commercial bone cement [42]. This ring-opening polymerization involved the growth of PDLLA on the hydroxyl groups of HA. 

Nano fibrous synthetic scaffolds with diameters in the range of 400 to 500 nm showing antibacterial activity against *S. aureus*, cell proliferation, in vitro degradation, and mineralization were fabricated by the electrospinning method using an 8 wt/v % solution of PCL, HA, and ZnO [64]. It was shown that ZnO may act as a triggering molecule in mineralization of the scaffolds [64]. 

Recently, a protein-based growth factor, bone morphogenetic protein 2 (BMP2), was successfully dispersed in PCL and poly(lactide-co-glycolide)-co-poly(etherimide) (PLGA-PEI) polyesters in the presence of soybean lecithin (SL) to form a bioactive osteo-polyester scaffold (BOPSC), thus avoiding the implantation of exogenous stem cells or osteoblasts [30]. The optimized BOPSC showed a porosity of 83.42%; BMP2 capture efficiency of 95.35%; water uptake ratio of 850%; and proliferation, migration, and osteogenic differentiation of mouse adipose-derived mesenchymal stem cells (mADSCs), which successfully stimulated natural bone regeneration after 6 months of implantation in mice.

**Figure 3:**
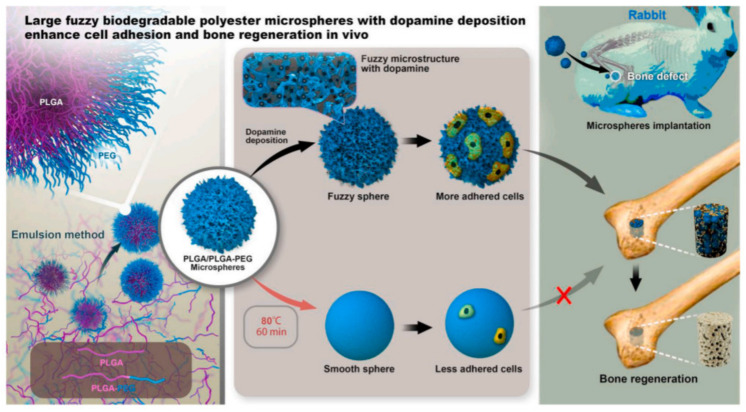
PLGA/PLGA-b-PEG microspheres obtained by interfacial instability of emulsion for bone adhesion in rabbit. Reproduced from [62] with permission from Elsevier.

Poly(D,L-lactide-co-glycolide) (PLGA)/PLGA-b-poly(ethylene glycol) (PEG) microspheres with a diameter in the range of 50–100 μm were obtained through a facile and controllable emulsion process following the mechanism of interface instability [62]. The ratio of lactide and glycolide in these two polymers was 50:50. A series of microspheres with different surface structures were further prepared through annealing and dopamine deposition, and their efficiency in bone regeneration was evaluated in vivo (Figure 3).

Porous scaffolds obtained using a conventional porogen-leaching technique, as well as TIPS methods, have many drawbacks, including the use of organic solvents and poor control of the shape and interconnectivity of pores, while electrospinning mats exhibit small pores that limit cell infiltration and tissue ingrowth. The 3D-printing technique is an innovative strategy that allows the development of hard-tissue engineering for bone regeneration. The introduction of 5 wt % or 10 wt % of β-TCP into PCL has a good effect on the thermal stability, crystallinity, and rheological properties of PCL composites, which are easy to process for additive manufacturing via fused filament fabrication (FFF) [65]. 

Innovative scaffolds with a three-dimensional (3D) architecture were obtained by 3D melt extruding of PCL with 20 wt % chitosan [66] and coating of 3D-printed PCL scaffolds with HA and BG [72].

Poly(glycerol sebacate) (PGS) is a polyester synthesized from glycerin-3 and sebacic acid-2 that has received considerable attention in tissue-engineering applications [80]. PGS was modified by 3D-printing technology with nano-HA [32], PEG/TCP [81], PHB [82], PCL [83,84], poly(vinyl alcohol) (PVA) [85], and poly(acrylic acid) (PAA) [86] to obtain potential scaffolds for reconstruction of bone tissue, especially craniofacial bone. Among the characteristics of an ideal polyester for bone regeneration, there is increasing interest in the development of novel materials with antibacterial properties [87]. Silver nanoparticles (AgNPs), graphene oxide (GO) sheets, and ZnO are reinforcing fillers used in polyester formulations for avoiding infections in the orthopedic field. The content of an antibacterial agent in polyesters should be optimized, as the materials do not have toxic effect. PLA/HA/AgNPs nanocomposites containing up to 10 wt % antibacterial agent are considered noncytotoxic [87]. In addition, the introduction of natural resources such as diatoms [70] into polyesters enhanced the bioactivity of bone-tissue engineering.

## 3. Tissue Engineering

### 3.1. Polyesters with Improved Functionalities

Natural and synthetic polymeric materials have gained a decent amount of attention in recent years for tissue engineering. The conventional material groups such as metals, metal alloys, and ceramics are still in use due to their undeniable superiority in the field of mechanical properties; however, polymeric materials are gaining an increasing amount of attention. Selected polyester materials that present a potential for producing scaffolds and different biomedical applications are summarized in Table 2.

Aliphatic polyesters such as PLA, PGA, PCL, and their composites have long been used for tissue engineering due to their good biocompatibility and biodegradability. Tuin et al. [99] reported a high-throughput process of (melt-blown, spun-bond, and carded) nonwoven manufacturing methods that were suitable for production of tissue engineering scaffolds from PLA. 

Electroconductive composites or blends have also found applications in tissue engineering, as reported by Wang et al., who prepared a series of electrically conductive nanofibrous sheets with similar fiber diameters for cardiac-tissue engineering [88]. Further, the authors utilized these conductive nanofibrous sheets to develop a series of cardiomyocyte-based 3D bioactuators with spontaneous contraction motion, here exemplified by the conductive PLA/polyaniline (PANI) nanofibrous sheets with different PANI contents prepared by the electrospinning technique [88]. The obtained results have proven to be promising for this type of application, as the investigated 3D bioactuator continued beating spontaneously with regular contraction patterns after 21 days of culturing. 

Cardiac-tissue-like patches are also an interesting approach, as reported by Cesur et al. [89], in which a randomly oriented PLA, PLA/PEG, and random and aligned PLA/PEG/collagen (PLA/PEG/COL) nanofiber patches were successfully produced by the electrospinning technique for myocardial tissue engineering, which is one of the promising treatment modalities for repairing damaged heart tissue in patients with heart failure. Interestingly, as reported, even a small amount of COL (1 wt %) in a PEG-plasticized PLA could increase the electrical conductivity by two orders of magnitude. Both randomly oriented and aligned fiber patches showed a therapeutic value in myocardial repair and tissue engineering, but the maximum cell viability rate was observed for aligned ones. Another interesting approach to treating cardiovascular diseases with surgical revascularization was presented by Jacob Hodge and Clay Quint, in which PGA-based scaffolds were investigated in terms of the response to a circumferential stretching on a tissue-engineered vessel obtained from an electrospun scaffold. What differentiated this work from other similar approaches was how the production of COL and cross-linking of COL fibers in relation to the mechanical properties of the engineered vessels was related to the mechanical stretching stimulation [92].

Research involving the use of PLA in a blend was investigated by Wrzecionek et al. [90], in which they synthesized poly(glycerol citrate) PGCit and further mixed it with PLA for creating porous nonwovens by electrospinning. The produced materials were tested for possible application in the field of tissue engineering. The addition of PGCit is a new approach; however, as authors pointed out, the obtained materials need further refinement before being extended to larger-scale medical applications [90]. 

The engineering of soft tissue as cartilage tissue was investigated by Chen et al. [100], who produced a 3D scaffold based on electrospun gelatin/PLA nanofibers. In addition, a process to cross-link a gelatin/PLA nanofibrous scaffold was realized by heating to a high temperature followed by water treatment (heat and water). The resulting scaffold exhibited a hierarchical cellular structure and a superabsorbent property [100]. The obtained materials had a relatively high compressive strength, and as tested, they could significantly repair the cartilage defects in rabbits. As stated before, different types of tissues can be grown using scaffolds. Thus, Grant et al. [10] proposed the first use of a sacrificial, transfected cell line to biofunctionalize an electrospun polymer scaffold for liver-tissue engineering. The authors decellularized the biofunctionalized scaffold and validated the platform using cells representative of the liver. Preliminary studies presented the development of a hepatic extracellular matrix–PLA hybrid composite that exerted a biological influence on liver cells, manipulating their microenvironment and resulting in alterations in their gene-expression profile, protein synthesis, and cell attachment and survival. Scaffolds for liver-tissue engineering manufactured from a PCL/chitosan (CS) composite were reported by Semnani et al. [94]. They produced the nanofiber by electrospinning. Subsequently, the mechanical properties, roughness parameters, regional anatomy, structure, hydrophilicity, and cell growth of epithelial liver mouse cells were considered for liver-tissue engineering. The measured properties were within specified limits, and the liver cells were completely infiltrated and attached to the scaffold after seven days. A similar approach was undertaken by Ghahremanzadeh et al. [95], in which the authors introduced two new galactosylation methods for modifying PCL/CS scaffolds. In the proposed procedure, chitosan powder was galactosylated and then blended with PCL, followed by the electrospinning technique. Two different methods were investigated: postgalactosylation treatment of already-produced PCL/CS by immersing the scaffold in the solution; and in situ galactosylation of chitosan performed simultaneously with a wet-electrospinning method [95]. An important area of the field of tissue engineering includes applications for bone-tissue recovery. A comparison between coaxial PCL-PLA/HA fibers and PCL-PLA scaffolds was investigated by Kareem et al. [101], in which the 2D and 3D PCL-PLA/HA scaffolds with core and shell structured fibers were produced using coaxial electrospinning. They reported that increasing the fiber alignment in the 3D scaffolds led to anisotropic mechanical behavior with reduced mechanical properties when tested across fiber orientations. The obtained structures showed a gradual reduction in their tensile properties after 12 weeks of immersion in simulated body fluid (SBF).

Composites based on PLA for tissue recovery were rarely investigated in recent years; however, Jiang et al. [91] proposed composite scaffolds with cubically interconnected pores fabricated using fused filament fabrication with two different types of iron-based particle fillers, including biocompatible stainless steel 316 L and pure iron. The 316 L steel was approved by the FDA as an implant material. According to the authors, the use of a specialized iron powder improved the dimensional accuracy and mechanical performance of the obtained scaffolds. The cell viability assay of the bone marrow cells cocultured with the 3D-printed scaffolds revealed superior cytocompatibility of the PLA/iron scaffolds compared with pure PLA scaffolds and PLA/316 L scaffolds. 

Apart from cardiac or skin-tissue engineering as described above, production of other types of tissues, such as nerves for treating peripheral nerve injuries, are currently being investigated. Dehnavi et al. [96] presented an electrospun conduit based on PGA/collagen/bioglass nanocomposites. Nerve reconstruction requires a specific approach, therefore in this study, the obtained PLA/collagen composite reinforced with BG was used as a material for producing a guidance channel that allowed axonal growth from the proximal to distal stump. 

The use of PGA-based scaffolds for manufacturing scaffolds in tissue engineering can be also applied in dental reconstruction, but not in terms of implants; rather, to endorse reconstruction of periodontal ligament tissue—specialized connective tissue that connects the cementum and alveolar bone. Wu et al. [102] investigated such application and found out that nonwoven PGA scaffolds provided effective structural support to promote the secretion of the extracellular matrix in periodontal (PDL) ligament tissue cells. Furthermore, the cell–PGA constructs implanted in nude mice formed such an engineered tissue with a well-developed blood supply. Thus, PGA scaffolds combined with PDL cells offer a strategy for complex periodontal tissue regeneration [102]. 

PHA-based materials present a broad range of mechanical properties, biodegradability, and biocompatibility that usually is obtained by microorganisms in a specific environment, depending on the level of nutrients. PHAs can be categorized based on two main criteria: firstly, based on the monomeric unit carbon atom number (3–5 carbon atoms—short chain length (scl; i.e., PHB), 6–14—medium chain length (i.e., poly(3-hydroxynonanoate) (PHN)), and 14 and more—long chain length); and secondly, based on their composition: homopolymers containing only one type of monomer unit, and heteropolymers composed of more than one type of monomer unit. Until now, the use of PHAs in tissue engineering was limited to hard tissues, mainly bone reconstruction [103,104,105]. This restriction was due to brittleness, a lack of bioactivity, a relatively high cost, and a slow degradation rate. Other applications have been quite limited to research purposes only. This is because, while genetically modified bacteria can provide a wide variety of modified polymers, the nature of microorganisms used (mainly inherited metabolic pathway) can result in end-product batch-to-batch variations, especially in terms of structure and composition. The efficiency of the production process with a low polymer concentration and high substrate cost must also be taken into account, as providing a large-scale production process is economically impracticable. Recent research in tissue engineering using PHAs has been mainly aimed at wound management [106,107], nerve regeneration [108], cardiac- and coronary-related tissue engineering, and bone reconstruction [49,105,109,110]. Due to relatively low market availability, only a few of more than 90 known PHAs are available. In terms of mechanical properties, the most elastic one is P4HB, while PHB is considered to be most rigid [111,112]. There are several papers describing the use of PHAs for soft-tissue replacement. Different methods of manufacturing PHA-based scaffolds were compared by Grande et al. [113]; namely, the conventional electrospinning of a PHB/gelatin/HA mixture and the combination of the electrospinning of a PHB/gelatin solution with the electrospraying of an HA dispersion. The obtained scaffolds exhibited a continuous cell proliferation, with a faster evolution of cell morphology for the gelatin-containing materials. A long-term biological investigation clearly demonstrated that the sprayed scaffold developed a high biomineralization rate [113]. Vascular tissue engineering involving the use of the PHB was investigated by Yao et al., in which they manufactured various films using solution-casting and electrospinning methods [97,113].

Another group of materials that is not discussed here but presents very promising results in terms of tissue-engineering applications are hydrogels, as they are quite similar to biological tissues in terms of their mechanical and chemical properties; present good water absorption, and most importantly, are generally biocompatible [114,115]. 

### 3.2. Manufacturing Methods

**Figure 4:**
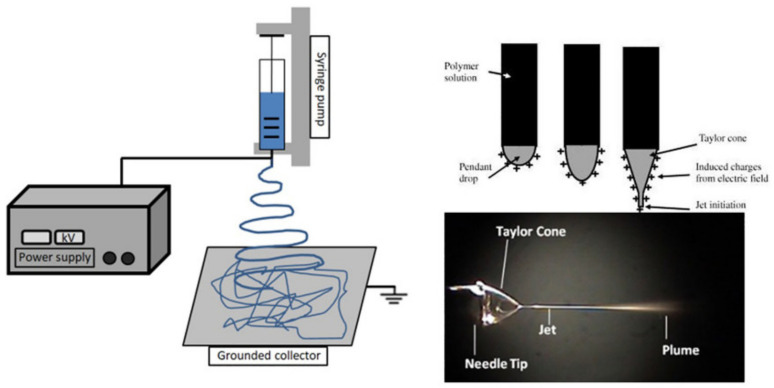
Schematic representation of an electrospinning device, showing the formation of the Taylor cone. Reproduced from [129] with permission from Elsevier.

There are various methods available for processing polyesters in tissue-engineering applications. Conventional processing techniques are available, such as compression molding [116,117,118], injection molding [119,120,121], melt spinning [122], and extrusion [123], along with other newer techniques, such as electrospinning and 3D printing, and others. For producing scaffolds in tissue engineering, electrospinning is one of the most promising methods [124,125,126,127,128], as it uses a polymeric solution passed through a stainless capillary and a high potential electric field between the nozzle and a grounded collector (plate shaped or a rotational drum), as presented in Figure 4. 

Producing a detailed structure with only a single layer of material can be time-consuming; however, such technique can be industrially upscaled by multiplying the number of layers being spun at once, resulting in a more complex mat structure and a faster production speed. Different factors influence the electrospinning process. The apparatus (applied electric field, distance between the needle and collector, and flow rate), solution (solvent, polymer concentration, viscosity, and solution conductivity), and environmental (humidity and temperature) parameters affect the fabrication of nanofibers [130]. Another relatively new technique used for producing scaffolds in tissue engineering is freeze-drying (or lyophilization), in which bioactive scaffolds with a porous architecture in planar 3D geometries are created. [131]. An interesting approach that combined those two methods was investigated by Chen et al. [93], in which electrospinning and freeze-drying processes were used for creating a superelastic scaffold with a cellular structure that consisted of nanofibers. The obtained scaffolds presented a porous structure, good water absorption capacity, and recyclable compressibility, while the cells seeded on them showed normal phenotypic morphology and proliferation. Salt leeching is a well-known technique still used for producing porous structures, including complex structures intended for tissue growth. This method works on the principle that a high-molecular-weight polymer solution in an organic solvent containing dispersed water-soluble salt particles is precipitated into an excess of nonsolvent. The polymer–salt composite is then processed by thermal processing methods into devices of varying shapes and sizes, and can subsequently be extracted to give the desired porous structures [132]. However, for polyesters, this method has its drawbacks, as they are prone to hydrolytic degradation. Investigating this subject, Xie et al. [133] reported a method to prepare biodegradable stereocomplex crystallite poly(lactide) (SC-PLA) porous scaffolds with high heat resistance, mechanical strength, solvent resistance, and biocompatibility by solvent casting and salt leaching. The resulted material proved to have a better biocompatibility and higher resistance to hydrolysis when compared to neat PDLA [133]. 

The 3D-printing methods, also known as additive manufacturing, have recently emerged on a global scale due to the decreasing equipment cost and more available and easier to operate software. One of the most common techniques is fused deposition modeling (FDM); others include stereolithography (SLA), selective laser sintering (SLS), and more [134,135,136,137]. The mentioned techniques, although time-consuming, have been shown to produce well-defined and reproducible structures that can be anatomically tailored to a specific case/patient. 

### 3.3. Specific Performances

Even though the first attempt to formalize the tissue-engineering discipline was published almost three decades ago [138], the field still garners a lot of attention from different scientific groups. As it is a multidisciplinary field, it involves doctors, materials engineers, chemists, and many other scientists. Therefore, it still is undergoing evolution with regard to the use of different types of biodegradable materials, with special respect to polymers and polyesters, which have a special part in this group due to their unique properties, including mechanical, biocompatibility, and biodegradation. Biocompatibility is of utmost importance, because a device intended to be used in vivo requires a lack of response from the patient’s immune system. Biodegradability, on the other hand, can guarantee that there will be no need for a second surgical procedure in order to remove the device (scaffold, patch, etc.). As presented, polyesters can provide a tunable set of properties that allow them to be tailored to a specific application, granting control over degradation time and other functional properties. Out of over 90 polyesters known to man, only certain types have found applications in the tissue-engineering field. In most cases, this is due to a wide variety of native tissues and their properties, where an ideal match between the tissue and the introduced material is not easy to find. However, progress in cell biology has given us a better insight into the living tissue, providing better knowledge on how to design scaffolds and other structures that can mimic the surrounding environment and stimulate cell growth without its disruption. In order to engineer a tissue in a patient’s body, the scaffold material needs to transmit the mechanical stimulation, as it is an important factor in stimulating the cell growth and final tissue development. In addition to matching mechanical properties, the selected material needs to be easily useable, must withstand the sterilization procedure, and must demonstrate low toxic (or preferably nontoxic) behavior at the cellular level. Furthermore, most of the presented studies that involved implementing a polyester-based structure in patient’s body investigated the expected sensitivities of the selected material to in vivo conditions, especially the environment’s pH, possible inflammation at the regional scale, and effects related to enzymes and protein adsorption. Continued material development will further increase the application potential of polyesters for tissue engineering. Recent developments suggest that more value-added materials are gaining the attention of researchers. For example, poly(glycerol sebacate) (PGS) was introduced into a polymer scaffold system, creating a self-healing structure that was responsive to environmental stimuli and capable of self-healing during degradation by providing constant structural support [98]. Another type of “smart” materials are those with a shape memory effect. A device made of such materials can be manufactured as a small, easy-to-implant element that will undergo specific changes in the patient’s body once it has been implanted due to the temperature changes in the surrounding environment [139]. The presented family of “smart” materials, including polyesters, have proven to have interesting applications, including as biodegradable medical sutures, scaffolds that become elastic, or stents that will expand once implanted [140,141].

## 4. Surgical Implants (Suture Materials, Tissue Adhesives, Surgical Meshes)

### 4.1. Tissue Adhesives

**Figure 5:**
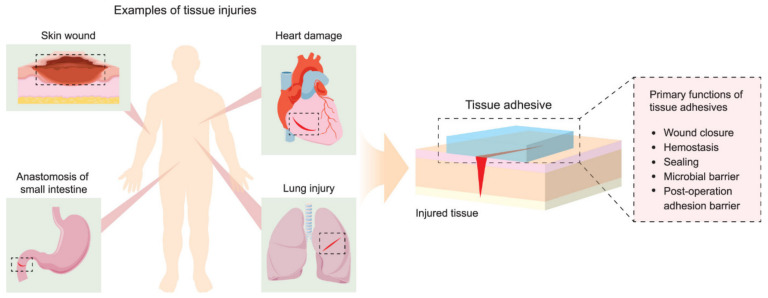
Examples of tissue injuries and primary functions of tissue adhesives. Reprinted with permission from [142]. Copyright 2021 American Chemical Society.

Other interesting applications for polyesters in medical implants include tissue adhesives, which must support materials that are able to promote tissue connectivity, and in some cases can reduce damage along with the healing process during the removal of the bandage or any other wound-covering mesh. The conventional approach to reconnecting damaged tissue for the time needed for a wound to heal involves use of sutures and staples. While the procedure of applying sutures is well known and provides an effective closure of relatively small wounds, it is time-consuming and requires appropriate equipment. Therefore, tissue adhesives are seen as a suitable alternative in providing wound dressings with an adhesive layer that must be in contact with the damaged tissue and a second layer consisting of the bulk polymeric network containing the adhesive [142]. The role of the adhesive layer is to keep the entire construction in place through chemical, physical, or covalent or noncovalent interactions. The adhesive matrix, on the other hand, provides structural support and determines the end properties of the adhesive in terms of stiffness, viscosity, swelling, abrasion, and degradability, as presented in Figure 5. 

Biodegradable polyesters such as PLGA or PCL have been investigated for such applications, as they are able to degrade completely into nontoxic compounds. In comparison, the nondegradable materials also used for the described applications, such as cyanoacrylates, isocyanates, aldehydes, and others that do not degrade, can cause an inflammatory reaction or release toxic byproducts. Other polyesters with the desired set of properties have been used to produce tissue adhesives, sutures, or meshes [143]. In order to better tailor the end material properties, copolymerization is often a common strategy. For example, a bioadhesive based on PCL functionalized with a low-viscosity, isocyanate-functional unsaturated acrylic ester [144] or 2-isocyanatoethyl acrylate (AOI) [145] that was manufactured into flexible transparent films with an interesting set of biomedical applications, more important for surgical adhesives, have been proposed. There are few similar UV-curable approaches that produce a tissue adhesive that can be applied quickly. Their potential is due to the requirements that such a material needs to have good interfacial contact with the wound sides, controllable solidification, and matching elastic moduli with the surrounding tissue [146]. Among other factors, the pressure application time also has an influence on the adhesion strength, as reported by Daristotle et al. [147]. 

### 4.2. Sutures

Sutures are well-known materials that join damaged tissues together. They should be biologically inert, with a repeatable set of properties; i.e., easy to handle by the surgeon, do not elicit harmful reactions, easy to sterilize, etc. From a medical point of view, the implemented suture should not promote microbial activity on the material’s surface. There have been some clinical studies regarding this issue that compared different commercially available sutures [148]. As reported, there were no significant differences between the selected materials (PET, silk, polyglactin, nylon), as all sutures were found to harbor bacteria, which in turn may have compromised healing of the surgical wounds. In order to create or generate the antimicrobial nature of a suture, it is essential that its surface be bioreceptive, allowing the biomolecules of certain bioactive components to anchor on the polymer’s surface. One of the most popular antimicrobial agents is Triclosan (2,4,4′-trichloro-2-hydroxydiphenylether), and studies of coating polyester sutures with this agent have been reported in the literature [149,150]. 

The ideal suture should have good mechanical properties, and should dissolve in the patient’s body after it serves its function by promoting tissue growth. Right now, the sutures commonly used can be divided into four main categories: absorbable, nonabsorbable, braided, and single filament [151]. Depending on the material used, absorbable sutures can withstand from around 10 days to 9 weeks. They can be manufactured from PLA (Polyglactin 910, Vicryl^®^, Polysorb^®^, Radik^TM^), PGA (Dexon^®^, Medifit^®^, Safil^®^) or other polymers from the PHA family (P(3HB), P(3HB-co-3HV)). In this case, the monitoring of mechanical properties over the course of in vivo degradation, as well as the correlation of mechanical properties with the provided application, are of high importance. Kehail et al. [152] investigated a copolymer P(HB-co-HHx) in vivo with a measured set of properties, and concluded that the material lost 58.5 ± 1% of its weight and 74.5 ± 2.5% of its Young’s modulus within 7 weeks. Apart from neat polymers, investigations of blends were also performed, as reported by Visco et al. [152], who developed a blend of PLA and PCL with ethyl ester l-lysine triisocyanate as a compatibilizing agent in order to explore its behavior in absorbable-suture applications. 

### 4.3. Surgical Meshes

Surgical meshes have represented a universal way to reinforce soft tissues since the late 1950s, when Dacron^®^ (PET) and Marlex^®^ (polypropylene, PP) were introduced. PP meshes are especially useful for hernia repair applications [153]. Meshes can be divided into four main categories, taking into consideration the component materials: nonabsorbable synthetic polymers (i.e., polypropylene), absorbable polymers (PGA, PCL), biologic (acellular collagen), or a composite material as a combination of the three previous categories. They have been continuously developed over the years, and given their composition, the type of the material, and the histological point of view, can be classified accordingly into: first generation (synthetic nonabsorbable prosthesis), second generation (mixed or composite prosthesis), and third generation (biological prosthesis) [154]. First-generation meshes are mostly based on PP systems, but polyesters such as PCL or PGA are also used (Dexon^®^); second-generation meshes were developed by combining more than one synthetic material in their compositions, mostly a combination of PP, PTFE, and some additives such as titanium (Ti) or poly(vinylidene fluoride) (PVDF). Third-generation refer to a biologic mesh material based on collagen scaffolds derived from donor sources. Their primary advantage is extensive promotion of the healing process combined with a nonexistent inflammatory response related to their excellent biocompatibility. However, due to the high cost of the third-generation meshes, their wide use is limited. A possibility to utilize a cheaper alternative for commercially available meshes was investigated by Todros et al. [155], who compared the properties of two multifilament industrial nests versus a surgical mesh (Parietex™ Lightweight Monofilament Mesh). All materials were made of PET polyester. Such an approach was dictated by the fact that similar materials are being used for hernia repairs in less-developed countries. The authors found similarities in the mechanical performances of industrial nets and patented surgical mesh, although further research concerning the response in vivo in biaxial stress conditions is required.

**Figure 6:**
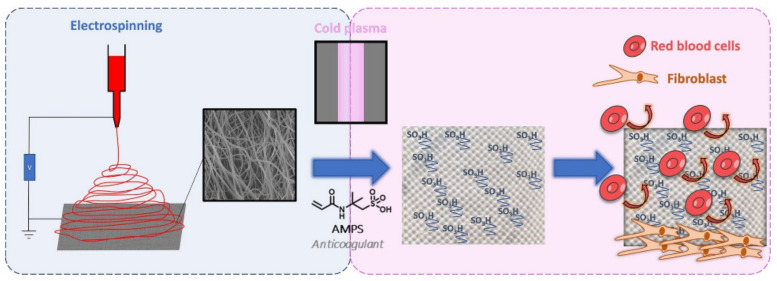
Schematic representation of the functionalization of PP mesh with PCL electrospun nanofiber monomer copolymerization. Reproduced from [160] with permission from Elsevier.

The human body’s response to foreign objects has been investigated, and according to [156], the mesh used for soft-tissue reinforcement may elicit a chronic inflammatory response that can be persistent over time. What also must be underlined is that the chemical structure of a polymeric mesh can be altered by the oxidative stress in biological tissues. According to [157], the structural changes in PP meshes can lead to crosslinking in polymer chains and formation of hydrogen bonds between the carboxyl groups, which can increase the mesh stiffness. Polyesters, on the other hand, are susceptible to hydrolysis, bulk or surface degradation, water uptake, and more, all of which influence their mechanical properties. In order to reduce the negative influence of meshes, different approaches have been investigated. Shokry et al. [158] developed a polyester fiber mesh layered with chitosan that increased the biocompatibility and increased the healing promotion for repairing abdominal wall hernias and prosthetics. Similar in concept but different in design was the investigation by Alin et al. [159], in which they proposed a polymer/nanotube coating on different surgical meshes, including polyester, by matrix-assisted pulsed laser evaporation. An electrospinning method for obtaining new surgical meshes is also being implemented (Figure 6). 

Dufay et al. [88] implemented a new method for covering a PP mesh with PCL electrospun nanofibers, although it was necessary to functionalize these polymeric nanofiber cover layers through an adequate surface treatment technique by cold plasma graft-copolymerization of the monomer (2-acrylamido-2-methylpropane sulfonic acid, AMPS) onto the surface of the nanofibers. The authors successfully grafted PCL fibers, and in terms of anticoagulant activity, the obtained samples with AMPS exhibited properties similar to that of 0.5 unit of heparin, without being affected by the plasma treatment and with acceptable cytocompatibility with fibroblast NIH3T3. 

## 5. Wound Dressings

### Manufacturing of Polyesters with Improved Functionalities

The compositions of some polyesters used for fabrication of wound dressings are shown in Table 3.

Many innovative polyester-based materials were developed in 2021 to meet the general requirements for wound dressings. Healing of wounds requires dressings with hydrophilic surfaces, which contribute to supporting cell adhesion better than hydrophobic surfaces. For example, CuO and ZnO NPs with contact angle values of 85° and 78° [20] and TiO_2_ NPs [18] were successfully used to increase the surface wettability of polyesters for wound applications. 

The PLA and PHB aliphatic polyesters are the most important bioresorbable polymer matrices used for development of wound dressings with excellent biocompatibility and biodegradability characteristics. They are bioresorbed by the body and act as a carrier for releasing of the bioactive compounds. The lactic acid oligomers containing carboxyl and hydroxyl end groups were quantified by EMI-MS tandem mass spectrometry as degradation products of P(D,L)LA/a-PHB and P(D,L)LA/a-PHB/proanthocianidins (PCAN) [161]. However, the neat PLA did not have good mechanical properties and bioactivity, having a great impact on the limiting of its applications in wound-healing management. The graft copolymerization technique has become a good strategy for chemical modification of lactic acid with cyanoacrylate directly in contact with tissue [165]; the study showed that the addition of graphene oxide (GO) up to 1% to a PLA solution for electrospinning enhanced its hydrophilicity, which positively influenced the drug-release kinetics [162]. Another study showed that the introduction of quercetin (Q), a natural flavonoid, could be fully released from PLA/GO electrospun scaffolds in just 1–2 min with the help of an external electric stimulus, showing an application for personalized wound healing [162]. Another model drug investigated for in vitro release was gentamicin sulfate loaded into PLA/poly(vinyl alcohol) (PVA) bioscaffolds [166]. 

PLA can be used as carrier for encapsulating bioactive compounds to design electrospun materials. For example, the antimicrobial agent cefazolin was encapsulated into a PLA–PEO solution as a core, while collagen was added to poly(vinylpyrrolidone) (PVP) as a shell to prove the accelerating of wound healing [16]. 

Coated melanin–TiO_2_/PCL/PHB mats allowed researchers to obtain a water absorption of 300 wt % [18], further supporting a key role for keeping the moisture balance in wound-dressing materials. The adding of gelatin to PHB nanofibers enabled them to obtain a wound-dressing material useful in treating second-degree burn wounds by decreasing the dressing frequency and the controlled released of silver sulfadiazine (SS) [24]. 

The inhibition of bacterial growth was reported in the case of copper oxide (CuO), zinc oxide (ZnO) nanoparticles, and tranexamic acid (TXA) added into PLA-coated sterile gauze [20]; silver sulfadiazine (SS) loaded into PBAT/PCL microfibers [22]; and neomycin (NEO), an aminoglycoside antibiotic, incorporated into carboxymethyl cellulose hydrogels [167]. Inhibitions of about 50%, 94%, and 95% were also observed in the case of PHB/gelatin/SS nanofibers against *S. aureus*, *P. aeruginosa,* and *E. coli*, and were assigned only to controlled release of SS [24]. 

A bioadhesive wound-healing gel prepared from a 30% gelatin solution coupled with poly(ethylene glycol) (PEG) and diethyl ether (DEE) as highly volatile solvents was shown to be an alternative to traditional first-aid dressings [17]. 

Electrospinning is a simple, low-cost, and scalable technique to prepare polymeric nano- and microfibers with different diameters, from microns to around 30 nanometers [161]. This method is based on using electrostatic forces to produce continuous polymer fibers from polymer melt or solution. Electrospun mats, due to their unique properties, great surface-area-to-volume ratio, and small pore size, are very beneficial in the absorption of body fluids, hemostasis, and the prevention of the penetration of bacteria. Additionally, the mats allow a good capability of cell attachment and proliferation, supporting skin wound healing. 

Nanofibers based on PBSu [15], PLCL/PCL [163], PLA/Hypericum perforatum oil (HPO) [21], PLA/GO/Q [162], PLA/silver (I)-diclofenac complex with (2-methylimidazole) [168], and PBAT/PCL [22] are few examples of polyester-based materials successfully obtained via the electrospinning method. 

The main conditions for processing of polyester-based materials via electrospinning method are shown in Table 3. The optimal conditions for obtaining free-bead fibers via electrospinning involve the setting of the flow rate, voltage, and distance between the needle and collector, as well as the working temperature and humidity, until a stable Taylor cone is formed. The choice of solvent is an important step in obtaining continuous and uniform fibers. The most common solvent mixtures used are chloroform:methanol, chloroform:ethanol, and dichloromethane (DCM):methanol at 90:10, 80:20, and 70:30 solvent ratios, respectively, for electrospinning of PBSu/food-grade agents [15]; DCM/N, N-dimethylformamide (DMF) at a ratio of 1:9 to obtain PLA/graphene oxide (GO)/quercetin (Q) electrospun scaffolds [162]; and DCM/DMF (7:3, *v*/*v*) and 1,1,1,3,3,3-hexafluoro-2-propanol (HFIP)/ethanol (7:3, *v*/*v*) for preparing encapsulated antimicrobial agents in PLA/PEO/PVP matrices [16]. The addition of acetic acid to the mixture of chloroform:ethanol to dissolve the PLCL/PCL copolymer prevented the formation of beads, and led to fibers with diameters in the range of 200 nm to 2.8 µm [163]. There were also reported nanofibers with diameters of 1.68 ± 0.58 µm and 1.51 ± 0.64 µm in the case of electrospun PLA/HPO [21], 1.107–1.243 µm in the case of PLA/GO/Q [162], and 1.9 ± 0.5 μm in the case of PHB/PCL modified with hybrid melanin–TiO_2_ nanostructures [18], which are considered as adequate for cell adhesion and attachment. However, the nanofibers’ average diameters of 599.94 ± 112.04 nm [16], or in the range of 250–300 nm [20], permitted the controlled drug release, antimicrobial activity, and acceleration of the wound-healing process. Thus, PHB/gelatin nanofibrous composition (70:30) with bead-free nanofibers, a uniform diameter, and a porosity of 55 ± 2.08% allowed the loading of a silver sulfadiazine (SS) drug [24]. The in vitro SS delivery revealed a burst release of ∼40% of the drug up to 5 h.

In addition, the thickness of electrospun nonwoven fabrics is an important parameter for air permeability favoring the healing of wound. It can be set from the volume of solution, as the thickness of the fabric can be ∼224 µm [21]. Innovative wound dressings made from renewable materials with proper mechanical properties were created by using a synthetic aliphatic polyester, PBSu, in the presence of a chain extender, diisocyanahexane (DCH), and natural food-grade components such as edible gums, essential oils, and free fatty acids by the electrospinning technique [15]. 

Wound dressings with adequate air permeability, water sorption, and good resistance against microbial organisms were also obtained by dip-coating of polyester/viscose samples in the presence of biogenic silver nano sol (AgNS) prepared using manuka honey [169]. In another study, poly(3-hydroxybutyrate) (P(3HB))/poly(3-hydroxyoctanoate-co-3-hydroxy decanoate) (P(3HO-co-3HD)) nanofibers were prepared via electrospinning, then an aqueous colloidal silver solution of 4000 ppm was used for dip-coating of nanofibrous scaffolds with dimensions of 30 mm × 60 mm at a rate of 170 mm/min to obtain antimicrobial wound dressings [170].

PCL is a synthetic degradable polyester that is highly hydrophobic, but without the ability to support cell adhesion and growth. This drawback is removed by co-polymerization with L-lactic acid, resulting in PLCL copolymers with certain applications in wound-healing management. For example, low- and high-molecular-weight PLCL masses were investigated as blend adhesives in healing, and were comparable with a polyurethane bandage [171]. Nanofibrous membranes based on PLCL/PCL and platelet lysate (PL) containing a mixture of various growth factors are a new therapeutic approach for wound healing to stimulate skin regeneration and the proliferation and differentiation of keratinocytes [163]. It was found that the composition with a 50:50 mass ratio of components showed a high adhesion strength, and was proposed for replacement of conventional bandage adhesives. 

The impregnation method of polyesters was employed for designing halloysite nanotube (HNT)-coated PET dressings to accelerate hemostasis [172], or loading with Annona leaf extract (ALE) for developing antioxidant and antimicrobial polyester transdermal patches [164]. The comparison of impregnation of ALE into a hydrocolloid sodium carboxymethyl cellulose (NaCMC) natural wound dressing and PD revealed that antioxidant activity was obtained the case of PD due to the swelling effect of the PD at supercritical solvent impregnation (SSI) conditions, which favored the large diffusion of the plant extract into the macromolecular chains of the polyester, and a high impregnation yield [164].

Weft-knitted spacer fabric production is a new advanced technology for developing 3D hydrocellular functional wound dressings from PET and PU yarn impregnated with 15 g L^−1^ of quaternary ammonium salt (QAS) solution [173]. This cost-effective construction showed a good property in moisture management and a broad-spectrum antimicrobial effect compared with conventional wound dressings based on carboxymethyl cellulose alginate, enzyme-based autolytic hydrogel, and polyhexamethylene biguanide dressings. Thus, the need for repetitive wound-dressing changes for diabetic patients could be reduced.

An increased attention to fabrication of personalized wound-dressing materials has been addressed to the 3D-printing (3DP) or additive-manufacturing (AM) technique. Fused deposition modeling (FDM)/fused filament fabrication (FFF) is one of the most common low-cost AM techniques. The designed model, microarchitecture, and geometry of wound-dressing materials were obtained by providing the successive layers of materials using appropriate computer-aided design (CAD) software or Digital Imaging and Communications in Medicine (DICOM) files [75]. 

In an attempt to reduce the effects of adverse antibiotics, modern wound dressings loaded with natural antimicrobial agents such as proanthocyanidins (PCAN) extracted from *Pelargonium sidoides* [161], Annona leaf extracts [164], babassu oil [19], Hypericum perforatum oil (HPO) [21], melanin [18], and arginine and chitosan [174] were developed. Immunomodulatory properties toward skin keratinocytes in vitro were related in the case of olive leaf extract (OLE) incorporation in poly(hydroxybutyrate-co-hydroxyvalerate) (PHBV) and PHB/poly(hydroxyoctanoate-co-hydroxydecanoate) (PHB/PHOHD) nanofibers, due to the antioxidant activity of oleuropein [175]. An antibacterial and antibiofilm dressing for wound healing was developed based on L-tryptophan and L-phenylalanine-based poly(ester urea)s electrospun fibers, which aimed at delivery of nitric oxide (NO), promoting proliferation, enhancing collagen, and accelerating wound treatment [176,177].

Kaempferol nanocrystals (KPF-NCs) loaded into a PHB/chitosan (CS) film could be another interesting blend for wound dressings, and had a remarkable blood clotting capacity for 20 min [178]. An accelerated improvement in wound healing was reported in the case of acrylate-endcapped urethane-based polymers (AUPs) prepared as films and electrospun mats [179]. In addition to showing the elimination of excess exudates and the provision of good mechanical properties, when these materials were analyzed in an acute wound model conducted in rats in vivo, they showed significant wound contractions as compared with positive controls.

Injectable hydrogels are a new class of wound healers that permit the moisture of wounds and oxygen permeability. Recently, boronic ester dynamic injectable hydrogels were developed as a novel self-healing, dual-stimuli-responsive, antioxidant, and antibacterial class of materials for healing of chronic wounds [180].

## 6. Vascular Applications 

### 6.1. Polyesters with Improved Functionalities

Vascular grafts are essential in the replacement of damaged blood vessels and the treatment of cardiovascular diseases. 

Transcatheter arterial embolization (TAE), a safe and innovative interventional therapeutic technique efficiently used for special vascular diseases treatments, consists of an embolic agent injection into a target artery [181]. The medical applications of TAE include: (a) treatment of vascular lesions (e.g., intracranial aneurysms and arteriovenous malformations) [181]; (b) supporting hemostasis [182]; (c) cancer treatment, including transcatheter arterial chemoembolization (TACE) for hepatic carcinoma [183]; (d) redistribution of blood (e.g., obstetrics and gynecology) [184]; and (e) promoting the surgical resection of internal tissue [185].

**Figure 7:**
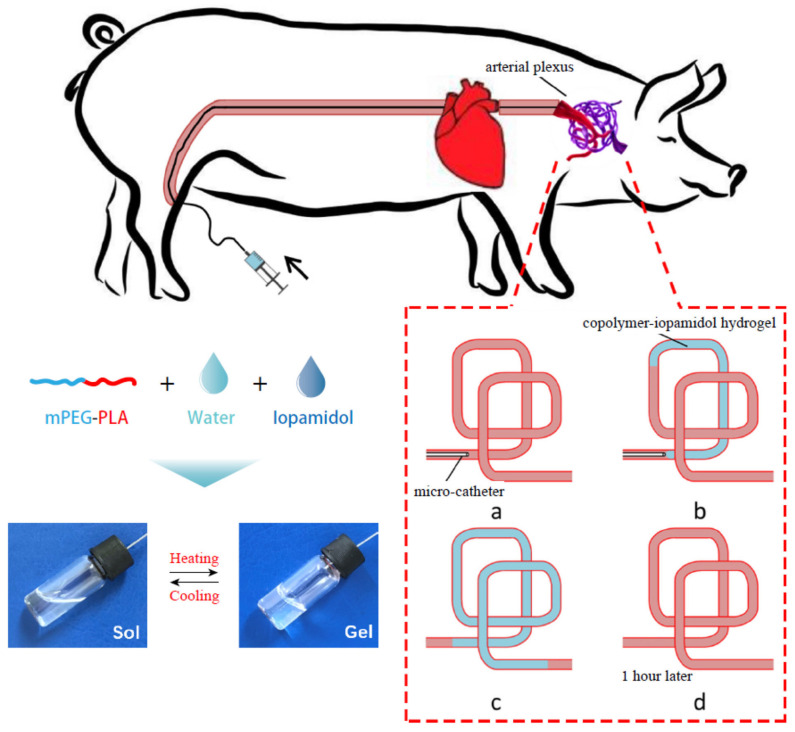
Schematic representation of the application of an mPEG–PLA thermogel as a temporary embolic agent for TAE. The aqueous mPEG–PLA solution containing iopamidol transformed from a free-flowing liquid at low temperatures to a gel when increasing the temperature (reversible sol-gel transition). Reproduced with permission from Elsevier [186].

Yang et al. [186] evaluated the efficiency of an injectable methoxy PEG-poly(D,L-lactide) copolymer (mPEG-PLA) thermogel in temporary vascular interventional therapy in the case of a large animal (swine). The results showed unique thermoreversible sol-gel transitions and superior injectability for PEG/polyester thermogels when compared with Onyx™, the only liquid embolic agent approved by the FDA so far. Figure 7 shows a schematic diagram for the in vivo use of mPEG-PLA thermogel as a temporary embolic agent for TAE in a swine model. The microcatheter was introduced and advanced in order to conduct the embolization in the neck of the pig (a); then an aqueous copolymer–iopamidol solution was injected, flowing along the blood vessels, and observed under X-ray exposure (b); successful embolization of the target artery was reached after microcatheter extraction, as at body temperature, the aqueous mPEG–PLA solution transformed to a semisolid gel, leading to temporary occlusion of the pharyngeal artery (angiography) (c); which was recanalized 1 h after the operation (d). The mPEG–PLA thermogel can thus be used as a potential temporary presurgical embolic agent for tumor resection, although extensive studies regarding the improvement in the mechanical strength of the gel are required in order to further enhance the embolization efficiency of the PEG/polyester thermogel.

Various synthetic vascular grafts have been used successfully, with clinical approval for medium to large vessels, while for small revascularizations, mainly autologous vascular grafts are approved at the clinical level. Due to qualitative and quantitative limitations of the latter, there is an enormous need to develop small-diameter (<5 mm) synthetic vascular grafts; therefore, new materials and innovative methods, such as decellularization, electrospinning, lyophilization, knitting, 3D printing, or even the combination of these approaches, have been considered [187]. 

Several examples of polyester-based materials used in vascular reconstructive surgery, along with their important features and specific applications, are presented in Table 4.

### 6.2. Risks and Limitations 

Peripheral vascular surgery creates a high demand for available small-diameter vascular grafts, but only a small range of newly developed materials have been successful in incipient experiments due issues such as acute thrombosis, incomplete endothelialization, and intimal hyperplasia after implantation. 

A limitation of complete studies involving the development of innovative polyester-based materials for vascular applications is the limited number of patients that can be subjected to experiments, out of which some could already have additional foreign materials in their organs/vessels. In addition, the risk factor for the new graft material is difficult to prove due to the diversity of vascular graft infections. Vascularization potential, reduced thrombogenicity, and secure pseudointima growth are key parameters that could affect the successful integration and functionality of prosthetic vascular grafts [200]. 

## 7. Ophthalmological Applications

### 7.1. Manufacturing of Polyesters with Improved Functionalities

Biodegradable formulations used in ocular treatments are generally manufactured using PCL, PLA, PGA, or PLGA [202]. Thermoreversible PLGA–PEG–PLGA triblock copolymers and modified chondroitin sulfate aldehyde formulations have been proposed as corneal adhesives [203].

PLGA is a widely used biodegradable polyester in the preparation of drug-delivery systems for various vitreoretinal diseases, such as age-related macular degeneration, uveitis, and diabetic macular edema [204]. Biocompatible and biodegradable PLGA and PLA are most widely utilized at the clinical level. The FDA approved a number of long-acting injectable (LAI) microsphere formulations due to their proven safety history. The selection of the PLGA/PLA in the design of microspheres depends on several factors, such as the specific administration route for a particular drug, the amount of microspheres distributed per dose unit, the daily rate of drug release from the microspheres in order to meet the therapeutic concentration of the specific drug, and the degradation time of the polymer. The injectability of the microspheres and drug-release behavior are influenced by the particle size of the PLGA/PLA microspheres [205]. 

One of the most common diseases in ophthalmology is eye inflammation, which can affect any part of the eye or surrounding tissues. The typical anti-inflammatory drugs used to treat ocular inflammation are corticosteroids, but their continued administration leads to serious side effects [206]. Nonsteroidal anti-inflammatory drugs (NSAIDs) are considered the principal alternatives to corticosteroids in the treatment of inflammations. New formulations of PLGA nanospheres (Nss) loaded with 0.5 and 1.0 mg/mL dexibuprofen (DXI) have been developed to upgrade the biopharmaceutical profile of the NSAIDs used for ocular administration [207]. A cell-viability analysis demonstrated the low cytotoxicity of PEGylated-PLGA nanospheres compared with free DXI. 

Micellar nanocarrier formulations based on methoxy-PEG-hexyl-substituted PLA (mPEGhexPLA) were well tolerated and nontoxic, efficiently delivering poorly soluble drugs to the anterior and posterior compartments of the eye in rats [208].

Intracameral PCL implants show the advantage of bypassing the corneal epithelial barrier and enhance the quantity of drug delivered to the target tissues, in contrast with topical administration. Compared to particles that can degrade more rapidly and can show burst release, the developed systems containing a PCL reservoir can present longer and continued zero-order release due to a larger drug payload, along with increased control over the diffusive polymer barrier [209].

Other polyester-containing systems loaded with various drugs that have been proved efficient in ocular treatments are presented in Table 5.

A 3D-printed device known as a “cornea-on-a-chip” that contains a clear polyester porous membrane separating four lower and four upper channels has been developed in order to move fluids over corneal cells, simulating the movement of tears over a blinking eye [217]. The movement changed the cells’ shape and increased the production of filaments that kept corneal cells flexible and elastic. 

### 7.2. Potential Risks

Besides the advantages of using polyesters in biomedical applications, particular risks have been observed in some cases and applications. In ophthalmology, clinicians need to take into consideration a probability of vision loss, especially in patients with a high risk of vascular issues. 

Several studies reported destructive ophthalmic complications as a result of injecting PLA as facial filler for cosmetic purposes. A case of loss of light visual perception in the right eye was reported for a 55-year-old woman 5 days after injecting cosmetic PLA filler in the right forehead [218]. Intraretinal hemorrhages and whitening, white intravascular thromboemboli widespread within the arcades, acute ischemia of the right optic nerve, and acute cerebral artery infarctions were observed, but there were no permanent focal neurologic deficits. PLA used as forehead filler could have been injected in the supratrochlear or supraorbital arteries, which are direct branches of the ophthalmic artery. Further, the filler emboli may be pushed in the internal carotid artery and then cerebral circulation. 

Wu et al. [219] described a case of a 49-year-old woman with various chronic diseases that experienced acute ocular pain and central visual loss due to retinal artery occlusion after injecting PLLA in the temporal region. Retinal whitening in the blocked vessels and optic disc edema were observed as a result of optic atrophy, with permanent vision loss in the patient being reported, despite of prompt use of a special topical treatment that included eye drops of brimonidine and hyperbaric oxygen therapy. PLLA is frequently used as effective subcutaneous filler, with results in long-term reconditioning of facial volume, because the PLLA microparticles promote the inflammatory response that stimulates collagen deposition in the extracellular matrix [220].

The evaluation of the pharmacokinetics after administration of new formulations is highly necessary. Although the PEG–PLA micelle system has shown generally good clinical prospects, the relationship between the oxidative state of the organism and the PEG-b-PLA administration has not been fully evaluated so far. Dvorakova et al. [221] demonstrated a potential health risk of PEG-b-PLA micelles that could cause neuroendocrine-disrupting effects.

## 8. Multiresistant Infection Prevention, including the COVID-19 Pandemic

### 8.1. Development of Antimicrobial, Antiviral, and Self-Sterilizing Materials Containing Polyesters

Due to the various factors that lead to the spread of antibiotic resistance, such as the use of multiple broad-spectrum agents; overuse of antibiotics in animal husbandry, human health, or aquaculture; or lack of efficient antimicrobial control, it can be estimated that by 2050, no effective antibiotic will be available if innovative drugs are not produced. Some alternative methods are being considered for controlling antibiotic-resistant pathogens, the most efficient being the use of essential oils (EOs), bacteriocins, antibodies, phage therapy, nanotherapy, or quorum-sensing inhibitors [222]. Antibacterial vaccines have recently become progressively important in managing bacterial infections and reducing the need for antibiotics. Novel nanoparticles have been designed to induce proper immune responses for effective antimicrobial defense [223]. 

Antimicrobial polyester materials with a functionalized PLA substrate were produced by using surface modification under γ-irradiation (doses of 10, 20, and 30 kGy), and emulsion-stabilization approaches. Two bioactive agents, namely clove essential oil and argan vegetal oil, were incorporated into chitosan, then immobilized on the surface of the functionalized PLA by a wet treatment involving carbodiimide chemistry [224]. Melt processing, another technique, was used by Darie-Nita et al. [225] to develop PLA-based antimicrobial and antioxidant materials containing bioplasticizers, as well as active agents such as vitamin E and cold-pressed rosehip seed oil encapsulated into chitosan by the emulsion method. 

Microbial infections also can be treated by using electrospun PLA mats loaded with *Thymus capitatus* (L.) essential oil (ThymEO) [226]. Specific characterization showed negligible cytotoxicity of the novel materials, while reductions in microbial viable cells were caused by both the liquid and vapors of ThymEO released from the mats. 

Antibiotic-free antibacterial polyester-based materials for biomedical applications were developed by incorporating various concentrations (1.5%, 3%, and 6% *v*/*v*) of peppermint essential oil (PEP) on PCL electrospun fiber mats with diameters of 1.6 ± 0.1 to 1.0 ± 0.2 μm [227]. The PEP loaded on the PCL fibers increased the wettability and antimicrobial activity against *Staphylococcus aureus* and *Escherichia coli* bacteria, and also improved the cell viability on electrospun fibers at all used concentrations after 48 h of cell culturing using normal human dermal fibroblast (NHDF), compared with the control.

Inorganic nanoparticles have shown great potential in antiseptic coatings and polyester-based materials to prevent pathogen transmission and infection with various viruses, including influenza, HIV-1, norovirus, and SARSCoV-2. Silver (Ag) nanoparticles presented exceptional bactericidal and virucidal efficacy against a wide range of viruses, and the potential for applications in self-sterilizing materials [228]. Demchenko et al. [229] developed new antimicrobial and antiviral nanocomposites based on PLA containing Ag nanoparticles of approximately 6.7 nm. The thermochemical reduction of the Ag+ ions in the presence of polyethyleneimine (PEI) was optimum at 160 °C for 5 min. The novel polyester-based nanocomposite (PLA–Ag–PEI) demonstrated strong antiviral activity against the herpes simplex virus type 1, influenza A virus, and adenovirus serotype 2, together with effective antimicrobial activity against *S. aureus* and *E. coli* strains [229]. 

The antiviral properties of silver nanoparticles incorporated in a PHA bioplastic film were tested against norovirus surrogates, murine norovirus (MNV), and feline calicivirus (FCV) by Castro-Mayorga et al. [230]. AgNPs at 0.27 ppm were homogeneously distributed within PHBV films by layering a coating of thermally postprocessed electrospun PHBV18/AgNP fiber mats over PHBV3 films obtained by compression molding. Virus inactivation was evaluated in cell cultures based on the cytopathic effects; the viruses were quantified by plaque assay at a 50% tissue culture infectious dose (TCID50). FCV was completely inactivated after 24 h of exposure at 37 °C, while MNV infectivity decreased by 0.86 log TCID50/mL. Entire inactivations of *S. enterica* and *Listeria monocytogenes* pathogenic bacteria were recorded after contact with the film in the same conditions. AgNP-induced denaturation of protein capsids and a synergic effect with nanoparticle-generated Ag^+^ ions that displaced essential bonds in the viral structure were proposed as possible mechanisms. Even at a low concentration, the AgNP incorporated in the PHBV film exhibited virucidal activity against norovirus surrogates, while negligible variations in optical and mechanical properties were observed. 

Copper oxide NPs also proved efficacy and low cytotoxicity, and therefore are preferred to be incorporated into self-sterilizing materials, including woven fibers and biodegradable antiviral polymers also used in the biomedical field. Novel antiviral biodegradable polymers based on electrospun PHBV enriched with 0.1% or 0.05% CuO nanoparticles were designed and examined by Castro Mayorga et al. [231]. The antiviral assay showed 1.83 and 3.19 log TCID50/mL reductions of murine norovirus infectivity for 0.1% and 0.05% nanoparticle films, while after 24 h at 25 °C, no infectious viruses were detected.

The potential of copper-based NPs for a self-sterilizing antiviral face mask was assessed by Borkow et al. [232]. The tested four-layer N95 mask contained two external spun-bond PP layers impregnated with 2.2% Cu_2_O NP (*w/w*): one internal melt-blown PP layer containing 2% Cu_2_O NP, and one polyester layer without copper-based nanoparticles. A potent inactivation of human influenza A was detected, as no infectious virions were recovered after 30 min exposure of the mask to H1N1 in a simulated breathing device. The mask’s filtration properties were not altered by the presence of Cu_2_O NPs within the mask fibers; on the contrary, five orders of magnitude were registered for the antiviral function of the copper-interwoven masks compared to the control N95 masks. 

### 8.2. Polyesters in Vaccines

Various polymeric nanoparticle-based vaccines against respiratory viruses have been evaluated in scientific studies. A study by Roth et al. on C57BL/6 mice demonstrated that a vaccine platform composed of a PEG-PLA nanoparticle hydrogel allowed codiffusion of hemagglutinin and a TLR 7/8 agonist adjuvant, leading to a sustained codelivery pattern [233]. Significantly higher antibody titers against the H1N1 influenza virus were recorded 56 days after vaccination. 

The benefits of using PLGA as a vaccine-delivery platform already have been justified. PLGA nanoparticles containing hemagglutinin with dual TLR ligands enhanced antigen-specific neutralizing antibodies against highly pathogenic H5N1 influenza and T-cell responses as compared to soluble antigens [234]. The antigen-specific memory of T cells was found to be persistent for 1.5 years after vaccination. The authors concluded that the proposed immunization protected completely against the lethal swine and avian influenza virus strains in mice, and induced strong immunity against pandemic H1N1 influenza in rhesus macaques.

The immunogenicity of PLGA nanoparticles encapsulating inactivated swine influenza virus H1N2 antigens (KAg) was evaluated in a pig model vaccinated intranasally with PLGA–KAg. The clinical results showed that the PLGA–KAg vaccine was highly effective in raising the mucosal immune response, a cross-protective, cell-mediated immune response that is stimulated against both H1N2 and H1N1 influenza [235].

An innovative viromimetic vaccine platform against the Middle East respiratory syndrome coronavirus (MERS-CoV) was produced by Lin et al. [236] based on a viral capsidlike hollow PLGA nanoparticle encapsulating an emerging class of stimulator of interferon genes (STING) agonist adjuvants. Immune activation and antigen recognition were facilitated by the rapid release of the adjuvant upon cellular uptake due to the acid-sensitive PLGA hydrolysis.

### 8.3. Sanitization 

Unfortunately, healthcare-associated infections occur, leading to patient suffering and increased healthcare costs. Person-to-person contact is considered the main infection route, but several studies have shown that environmental surfaces also serve as important pathways of nosocomial pathogen transmission [237]. Patient privacy curtains surrounding beds in hospital are high-touch surfaces that can retain and spread bacteria; therefore, they require efficient cleaning in order to avoid pathogen transmission. The presence of methicillin-resistant *Staphylococcus aureus* (MRSA) and microbial contamination were determined on 10 polyester/cotton curtains freshly laundered and exposed in a regional burns/plastics unit of a hospital. Microbial contamination was present right after day 3, while 1 out of 10 (1/10) curtains tested positive for MRSA by day 10, and 5/10 by day 14, exceeding 5 colony-forming units (CFUs)/cm^2^ by day 21. These results showed that curtains are a source of cross-contamination in hospitals, and interventions for effective cleaning or replacement should take place approximately 14 days after their initial hanging [238].

The microbial barrier of several polyurethane/polyester fabrics containing knitted polyester fabric in the substrate used in surgery and for wrapping sterile items was evaluated following the action of washing and sterilization under real hospital conditions [239]. The highest shrinkage that stabilized after 10 washes was recorded for the knitted polyester samples, compared to the sample with a knitted polyamide fabric that contracted less but needed more washing and sterilization cycles to achieve dimensional stability. After 0, 10, and 20 washing and sterilization procedures for one, two, and three months, all tested samples showed an effective microbial barrier and durability.

### 8.4. Polyesters Used against the COVID-19 Pandemic

Prevention of viral infections can also be achieved by fast identification and isolation of infected patients; therefore, a large number of tests must be performed. In this regard, there is a widespread demand for multiple nasopharyngeal swabs. Polyester swabs are cost-effective and can be produced at a higher capacity compared with foam swabs, and have a similar performance in nasal collection, so the FDA considers polyester swabs to be acceptable for SARS-CoV-2 testing [240]. In addition, when compared with cotton or rayon swabs, polyester swabs proved a higher absorption capacity and extraction efficiency for retrieving human DNA from salivary samples [241]. Padgett et al. evaluated the stability and performance of several types of polyester nasal swabs, including the Fisherbrand polyester-tipped applicator (Fisher Scientific #22-363-170), SteriPack spun polyester (SteriPack #60564 and #60567), and Copan spun polyester (Copan Diagnostics #164KS01) stored in dry collection tubes, and discovered their identical efficiency with foam swabs in SARS-CoV-2 detection, with the viral RNA remaining stable under home self-collection and cold- or warm-expedition conditions to the laboratory simulated by two freeze–thaw cycles or 72 h at a high temperature. RNase P detection demonstrated that sufficient material for molecular testing was collected by using these types of polyester swabs [242]. A prototype of a low-cost PLA nasopharyngeal swab produced by 3D printing (USD ~0.05 per swab) and tipped with polyester proved to have a higher sensitivity (90.6% versus 80.8%) in detection of SARS-CoV-2 in comparison with commercial swabs when tested on almost 300 patients [243]. In order to confirm the achievement of the desired mechanical properties required for a successful maneuver through the nasal cavity, 3D-printed PLA swabs were subjected to a 180° bend test, and proved they had the necessary flexibility–rigidity balance. Considering that the glass transition temperature of PLA is ~60 °C, when the polymeric material softened, hydrogen peroxide plasma sterilization of the 3D swab prototype was performed at 45 °C. 

Recent studies showed the presence of anti-SARS-CoV-2 IgA in ocular secretions of patients infected with SARS-CoV-2, confirming the conjunctival route of infection, as conjunctival tissue is an optimal site for virus replication [244]. Two possible routes of transmission have been suggested; namely: tears flowing to the face surface reaching the nose, and blood flow from the eyes or tears flowing to the respiratory system through the nasolacrimal ducts, even if obstructed [245]. Mahmoud et al. [246] used a polyester fiber rod sized at 2.0 mm × 10.0 mm (Transorb Wick, Filtrona, Richmond, VA) to collect tears from the lower fornix of the eye, and demonstrated that ocular symptoms were moderately correlated with a high IgA titer and greatly linked with reactive results of IgA. No correlation with age, sex, or severity of the disease was found.

A commercial 3D conductive filament of carbon black and PLA was used in the development of a special electrochemical immunosensor [25] that proved its efficiency for quantitative detection of theAraucaria hantavirus nucleoprotein (Np) and virus detection in human serum samples (100× diluted). The polyester-containing immunosensor could covalently anchor biomolecules, showing promise in the future detection of SARS-CoV-2.

Based on a study that assessed the stability of two viruses, namely SARS-CoV-2 and SARS-CoV-1, in different environmental conditions such as aerosols, plastic, copper, cardboard, or stainless steel, researchers concluded that SARS-CoV-2 would have a short life on copper and cardboard, and would be more stable on plastic and stainless steel [247]. De Albuquerque et al. [26] suggested that PLA combined with copper as an antimicrobial agent would be a potential material in the production of PPE. Polyesters are among the polymers used in the manufacture of disposable face masks, along with PP, polyacrylonitrile (PAN), PU, polycarbonate (PC), polystyrene (PS), or high-density polyethylene (HDPE). Usually, these masks comprise three layers, including an inner layer made of soft fibers, a middle layer containing a melt-blown filter, and an outer layer composed of water-resistant unwoven fibers [248]. Reusable cloth masks are mainly made of polyester or cotton–polyester mats. Microfibers are easily released from this type of mask, identical to the extraction of polyester fibers from textiles during washing [249]. At the beginning of the pandemic, Ahmed et al. designed a reusable, custom-made, recyclable face mask with special antimicrobial and antiviral properties suitable for large production that contained a filtration system produced by electrospinning based on a nanofibrous membrane of PLA and cellulose acetate with copper oxide nanoparticles (CuONPs) and graphene oxide nanosheets [250]. The inhibition ability of CuONPs on the growth of fungi, viruses, and bacteria has been already proved [251], as well as their antifouling properties, which prevent the adhesion of pathogenic microbes in the material containing them [252]. He et al. [253] produced biodegradable disposable filters for face shield masks by combining PLA nanofibers obtained by electrospinning with 3D-printed PLA filaments. The nanoporous filter allowed easy breathing and was highly useful for persons with hearing impairments, as it allowed lip reading due to transparency.

Surgical face shields are generally made of PET and PC foils, as both polymeric materials are transparent and lightweight, provide high optical clarity, and can be easily processed in different shapes, allowing the design of shields and masks that cover the requirements of complex applications. The transparent sheets of face-shield visors typically contain polyester. DuPont Melinex^®^ FS1 presents antifog properties that are essential for face-shield visors, and is available in sizes of 175 µ and 250 µ. The application of the face-shield visor was set at 175 µ to realize a perfect balance between transparency and stiffness, so Melinex^®^ FS1 successfully covered the required EN 166 standard and received the CE mark, and is being used in the UK NHS and other countries in Europe and Scandinavia [254]. 

In order to help otolaryngologists overcome dangerous exposures to respiratory droplets and aerosols during nasal and oral examinations, when coughing and sneezing in patients with COVID19 infection often occur, two researchers designed and produced a 3D-printed adapter for medical headlights that can hold a transparent sheet [255]. PLA was selected as the material used for 3D printing, as it is commonly used and cost-efficient; the resulting adapter weighed only 7 g, and had an estimated price of USD 0.15. The scientists used easily removed transparent sheets made from polyester for laser printing, with a cost of about USD 0.40 per unit. The resulting polyester-based prototype was successfully used in seven different headlights.

Polyester, PP, nylon, and rayon are synthetic fibers contained in nonwoven fabrics such as wet wipes, which are considered as PPE against COVID-19 [256]. Seventh Generation Inc. (USA) uses polyester spun lace in the manufacturing of different types of PPE, such as wipes, masks, or medical gowns [257], items that contribute at their end life to the growing plastic waste contamination due to COVID-19, and require immediate attention. Lee et al. found that polyester represents the major component of microplastics released in fiber form from wet wipes [258]. In the case of aquatic environment exposure, 693–1066 particles (p)/sheet polyester microplastics were released, compared with 180–106 p/sheet when rubbing the wet wipe on solid materials, with most of the fibers (>90%) having a size of more than 100 μm.

Excessive use of disposable face masks has produced large amounts of plastic waste, which, together with the release of MP, has led to environmental contamination, and the majority of the materials biodegrade slowly, or are non-biodegradable. In a study regarding the ubiquitous PPE pollution in South America, which has been exacerbated by the persistence of the COVID-19 pandemic, De-la-Torre et al. [259] mentioned that face masks, gloves, and face shields were the most common polluting forms of PPE, and used analytical methods (FTIR spectroscopy, SEM-EDX, XRD) to elucidate the structural and chemical changes of the PPE in the marine environment. PET and nylon were found to be the main components of the elastic cords in two face masks, similar to the report by Shen et al. [27]. Latex and PET were the main components of gloves and face shield visors. Various metals were also found in the analyzed PPE waste, including Ti, which is considered to be derived from TiO_2_, a common additive in polyester fibers [260].

**Figure 8:**
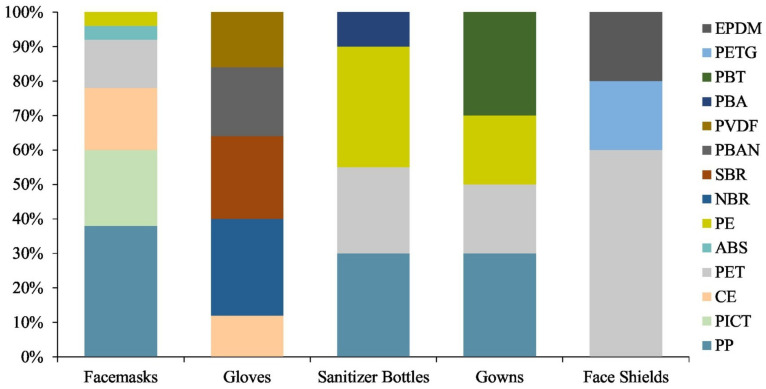
Composition of polymers in the selected commercially available PPE [261]. Reproduced with permission from Elsevier.

Robin et al. analyzed the types and amounts of plastics contained in biomedical wastes in India, and found an increase of 17% due to the COVID-19 pandemic in 2021. FTIR-ATR analysis revealed 25.4% PP and 15.4% polyester in the studied types of PPE [261]. The authors identified 14 polymer types in the evaluated PPE samples. Polyesters such as polybutylene terephthalate (PBT), PET, poly(ethylene terephthalate glycol) (PETG), poly (1,4-cyclohexanedimethylene isosorbide terephthalate) (PICT), and poly(butyl acrylate) (PBA) were mentioned, along with other polymers, namely acrylonitrile butadiene styrene (ABS), cellulose (CE), ethylene propylene diene monomer (EPDM), nitrile-butadiene rubber (NBR), polybutadiene acrylonitrile (PBAN), polyethylene (PE), polypropylene (PP), poly(vinylidene fluoride) (PVDF), and styrene–butadiene rubber (SBR). Figure 8 presents the types and amounts of polyesters and other polymers found in the commercial PPE.

Thermal processing by incineration or pyrolysis can be considered a major approach in the waste management of the increasing amounts of used disposable face masks. Ali et al. reported essential characteristics that govern the thermal decomposition of recognizable plastic-based components in 3M N95 face masks [262]. PP, the constituent of the protective three or four layers of the evaluated masks, had one step of degradation in the 330–480 °C temperature range, while decomposition of the polyester ear straps of surgical and N95 masks led to char residues of 24% and 15% fractions of the initial mass, respectively. This investigation imparted valuable information such as potential emission profiles or thermal stability regions for containing polymers in face masks used during the COVID-19 pandemic, potentially useful for safe and economic recycling of extensively used disposable masks or other types of PPE waste.

## 9. Conclusions and Perspectives

Polyesters have a well-established position in the field of medical applications. Based on their properties, they can be an alternative to other polymeric materials. They have been used as implants for decades (since 1958), and were one of the first polymers used for such applications. They present excellent mechanical properties along with an inert nature (i.e., PET), and some of them are biocompatible and absorbable, predominately due to their natural origin. PLA, PHB, and PCL polyesters cannot be used as a neat form for medical purposes, due to the lack of hydrophilicity and bioactivity. Therefore, implantation in the human body is an obvious choice, with the ability to tune the end properties of a material by copolymerization or by other means of combination with other substances, such as biologically active additives.

Polyester composites are promising biomaterials in various medical applications, and their improved properties have led to progress in bone-replacement materials and bone regeneration. Other polyesters are required to be used in longer-load orthopedic applications. The new trend in polyesters used in orthopedic applications is to develop a new polyester class based on the valorization of monomer byproducts. For example, aconitic acid/glycerol (AG) polyesters were synthesized from sugarcane industry byproducts [73].

Conventional wound dressings in the form of gauze, lint, plasters, and bandages [263] have not resulted in proper wound-healing management. The 3D-printing technology used to obtain films, foams, hydrogels, hydrocolloids, and smart wound dressings (including stimuli-responsive wound dressings, self-healing wound dressings for motional wounds, and self-removable wound dressings), is a challenge in this emerging field [264]. However, further research aims to extend the antimicrobial studies using a wider spectrum of pathogenic organisms, including common opportunistic fungi. In vivo studies in animal models will better contribute to understanding the healing properties of these polyesters when they are in direct contact with wound fluids, blood, and immune cells. 

Future work should focus on polyester-based scaffolds in large animal models, as well as in human clinical trials. The development of custom-made 3D composite scaffolds that can be grafted directly with stem cells in clinical practice is a promising approach. With this in mind, and considering the ongoing development in the field of large-scale production by 3D printing or electrospinning, there is a high potential to implement such structures in common clinical procedures. This will, however, require a close collaboration between the material engineers, designers, and end users to establish an appropriate design and the end criteria that they will need to follow. 

Future alternative methods proposed for prevention of multiresistant infections might be successful if used in combination with available antibiotics, without completely replacing them. The competent authorities need to encourage and accelerate this by providing appropriate financial support to promising studies regarding the development of safe alternatives for reducing the transmission of viral infections.

Optimally designed polyester-based materials and unlimited methods have yet to be explored within specific utilization targets due to the complex functionalities that need to be achieved for their biomedical applications.

## Figures and Tables

**Table 1:** Examples of modified polyesters obtained by different techniques for bone-tissue applications.

Composition of Polyesters	Method	Features/Remarks	Application	Ref.
HA/PDLLA/stannous 2-ethylhexanoate (SnOct_2_) nanografts	“Graft-from” polymerization	Improved mechanical properties of commercial bone cement	Bone cement applications by mixture with commercially available poly(methylmethacrylate) (PMMA)-based bone cements	[42]
PLGA and β-TCP porous structure	A modified solvent-merging/particulate-filtering method	The defect created within the rabbit femur was filled with new bone in 3 months	Bone substitute	[59]
PLGA scaffolds with cell-laden, platelet-rich plasma (PRP) hydrogels	3D printing	Proper mechanical properties and delivery of mesenchymal stem cells (MSCs)	Articular cartilage and subchondral bone within osteochondral defects	[61]
PLGA/PLGA-b-poly(ethylene glycol) (PEG) microspheres	Interfacial instability of an emulsion	Adherent bone-marrow-derived mesenchymal stem cells (BMSCs), A549, and MC 3T3 cells	Repaired femoral condylar bone defects in rabbit at 12 weeks post-surgery in vivo	[62]
PLGA-coated, vancomycin-loaded silicate porous microspheres	Coating	Cytocompatibility MTT assay	Drug delivery, bone-tissue engineering, and dental bone grafting	[63]
PCL/HA/ZnO nanofibrous structures	Electrospinning	Mimic extracellular matrix of immature bone; lower cell proliferation as compared with PCL and higher mineralization	Mid- and long-term resorption of bone	[64]
PCL/SiO_2_ aerogel composite material	Solvent casting	Good biocompatibility	Bone scaffold material	[35]
PCL/Poly(lactide-co-glycolide)-co-poly(etherimide) (PLGA-PEI) polyesters/soybean lecithin (SL)/bone morphogenetic protein 2 (BMP2)	Solid–liquid phase separation	Allogeneic bone formation after 6 months of implantation in mice	Repair of non-load-bearing bone	[30]
PCL/nHA/poly(ethylene phosphoric acid) (PEPA)/vancomycin mats	Electrospinning	Drug release against *S. aureus*	Bone surgery and orthopedics	[60]
PCL/TCP filaments	Fused filament fabrication (FFF)	5 wt % and 10 wt % of TCP in PCL are optimal for mechanical properties and controlled geometry of the FFF process	Guided tissue regeneration	[65]
PCL/chitosan scaffold	3D melt extrusion	The most hBMSC growth, swelling, and minimal degradation after 28 days as compared with PCL/TCP	Bone repair	[66]
Poly(butylene succinate) (PBSu)/sebacic acid (SeA)/magnesium compound membranes	Copolymerization	Excellent cell adhesion and cell proliferation of MC3T3-E1 (murine pre-osteoblasts) and L929 (murine fibroblasts) at 24 h and 48 h; bone-regeneration rate in rats implanted with bioactive polyesters after 16 weeks of experiment; the degree of inflammation was lower as compared with commercial PLA film	Guided tissue regeneration	[31]
PBSu/silica nanotubes or strontium HA (Sr-5(PO_4_)(3)OH) nanorods	Melt mixing	High enzymatic hydrolysis rate	Tissue engineering	[67]
Poly(butylene succinate) (PBSu)/strontium HA nanorods (SrHA nrds)	In situ polymerization	Nanocomposites showed high hydrolysis rates and biocompatibility, and promoted the formation of HA on the PBSu surface	Tissue engineering	[68]
Poly(glycerol sebacate) (PGS)/nHA microporous composite scaffold	Thermally induced phase separation (TIPS); thermal cross-linking (TCL) and salt leaching (SL)	Production of IL-1 beta, IL-6, and TNF-alpha osteoclastogenic cytokines	Reconstruction of bone tissue	[32]
PHBV copolymer porous films (content of HV varied from 0 to 12 mol%)	Solvent casting	The high content of HV led to a mass loss of 9.2% upon 19 weeks of exposure to pH 7.4 PBS and proliferation of osteoblast cells; A stability up to 7 weeks at pH 7 and temperature of 37 °C	Orthopedic surgical implants	[33]
Poly(3-hydroxyoctanoate-co-3- hydroxydecanoate) (P(3HO-co-3HD)); Poly(3-hydroxybutyrate) (P(3HB)); P(3HB)/P(3HO-co-3HD) 80:20; P(3HB)/HA fibers	Pressurized gyration	Increased DNA content with time, evidence of cell attachment and proliferation on the fibers; P(3HB) and P(3HB)/HA composite fibers demonstrated excellent potential as scaffolds to support and enhance bone formation	Scaffolds for both hard (bone) and soft (nerve and cardiovascular) tissue regeneration	[34]
PHB/BC scaffolds	Salt leaching	Good biocompatibility; increased osterix (OSX) and enhanced alkaline phosphatase (ALP) activity in the first 4 weeks postimplantation in adult CD1 mice; New bone formation after 20 weeks postimplantation	Larger bone defects	[69]
PHBV/PCL-pullulan (core)/diatom scaffold (DS) (shell)	Co-electrospinning	Controlled release of cefuroxime axetil (CA); improved human osteosarcoma (Saos-2) cell viability;	Bone-tissue engineering	[70]
Mesoporous bioactive glass nanoparticles (MBGN)/cinnamaldehyde (CIN)/(PHBV	Emulsion solvent extraction/evaporation	antimicrobial activity against *S. aureus* and *E. coli* Human osteosarcoma cell (MG-63) cell proliferation and attachment; rapid HA formation in simulated body fluid (SBF);	Antibacterial biomaterial for bone-tissue engineering	[71]
Self-made polyester-urethane filament	Fused filament fabrication (FFF)-based 3D printing (3DP)	Tensile strength ~30 MPa, Young’s modulus ~0.2 GPa, and compression strength ~1.1 MPa; It is stable for up to six months of incubation in 0.1 M PBS,	Cancellous-tissue engineering	[51]
PCL, PCL/HA, PCL/BG, and PCL/HA/bioactive glass (BG) scaffolds	3D printing by fused deposition modeling (FDM)	Cell adhesion and proliferation, and efficient potential in inducing osteoconduction and osteointegration as compared to PCL alone; PCL/HA/BG scaffold exhibited higher in vitro cell viability and bone formation	Bone-tissue engineering applications	[72]
Aconitic acid–glycerol (AG) polyesters	One-step polycondensation	Highest levels of mineralization, increased alkaline phosphatase (ALP) expression, and the greatest osteocalcin (OCN) expression after 21 days compared to PCL/HA (control)	Potentially bone-defect repair	[73]
Urethane-based PEGylated poly(glycerol sebacate) (PEGSU) in ceramic bioink bioscaffold	3D printing	New bone formation in critical-sized cranial defects	Atomically scaled craniomaxillofacial bone structures	[74]
Thermoplastic poly(ester urethane (PUR)/PLA loaded with amikacin sulphate antibacterial composition	3D printing FFF	Antimicrobial activity against *E. coli*, *P. fluorescens*, *S. aureus*, and *S. epidermidis* bacteria	Bone and cartilage (e.g., septum implants) scaffolds	[75]
Poly(glycerol-co-sebacic acid-co-l-lactic acid-co-polyethylene glycol) (PGSLP) scaffold filled with gelatin	Thermally induced phase separation (TIPS)	Local release of deferoxamine (DFO)	Vascularized bone regeneration	[76]
Polybutylene-adipate-terephthalate and niobium-containing 30 wt % BG (PBAT/BAGNb) composite	Casting	Increased proliferation and mineralization	Guided bone regeneration	[77]
Poly(ester amide) (PEA)/BG hybrid microparticles	Sol-gel	Bioactivity and dual drug release	Bone regeneration and therapy	[78]

**Table 2:** Properties of selected polyester materials and composites for tissue engineering.

Material	Mechanical Properties	Mechanical Testing Parameters	Applications	Ref.
PLA	σ_max_ = ~10 MPa	3D-printed samples, tensile rate 50 mm min^−1^, room temperature	Soft-tissue scaffolds	[6,10]
PLA/PANI	Displacement of 4–7 µm	Electrospun polymer blend network plus cardiomyocytes (CMs)	Cardiac-tissue regeneration	[88]
PLA/PEG/collagen	σ_max_ = 0.11–5.9 MPa ε_max_ = 72–84	Tensile rate 5 mm min^−1^, room temperature	Cardiac-tissue regeneration	[82,89]
PLA/PGCit	σ_max_ = 2.9–5.6 MPa ε_max_ = 53.7–103.7	Tensile rate 5 mm min^−1^, room temperature	Soft-tissue scaffold	[90]
PLA/iron	σ_max_ = ~20 MPa	Flexural tensile rate 1 mm min^−1^, room temperature	Bone-tissue engineering	[91]
PGA	σ_max_ = 1.86 MPa E = 7.62 MPa	Electrospun, tensile rate 0.5 mm s^−1^, room temperature	Vascular graft	[92]
PLA/GEL	σ_max_ = ~0.5 MPa	Electrospun, strain rate of 5 mm min^−1^, compression at ε = 80%	Soft-tissue scaffolds	[93]
PCL/chitosan	σ_max_ = 1.27–1.43 MPa E = 7.2–7.8 MPa	Tensile rate 15 mm min^−1^, 37 °C	Liver-tissue scaffolds	[94,95]
PGA/COL/bioglass	σ_max_ = ~8 MPa	Electrospun	Nerve regeneration	[96]
PHA/P(3HO)/P(3HB)	σ_max_ = 1.4 MPa E = 35 MPa	Tensile rate 10 mm min^−1^, room temperature	Nerve regeneration	[28]
PHB, PHBV	σ_max_ = 18.44; 18.68 MPa ε_max_ = 0.81; 1.01	Electrospun vs. casted	Vascular-tissue regeneration	[97]
PGS	σ_max_ = ~ 2 MPa ε_max_ = 0.5–2.5 E = 1.88–2.63 MPa	Tensile rate 125 mm min^−1^	Soft-tissue regeneration	[98]

**Table 3:** The main technological parameters and performance for polyester-material-based wound dressings.

Composition	Method	Technical Conditions	Features	Ref.
(P(D,L)LA/a-PHB) 70/30 wt %/proanthocyanidins (PCAN) 20 wt %	Electrospinning	10% *w/v* solution of polymers in hexafluoroisopropanol (HFIP) as solvent; voltage power of 21 kV; distance between collector and needle of 20 cm; flow rate of 1.5 mL/h; temperature of 25 °C; relative humidity of 27%	*Tg* value was 37 °C; 50% of PCAN was released during the first 10–12 days due to the diffusion mechanism; hydrolytic degradation of fibers occurred between 10 and 85 days; cytocompatibility with the tested cell lines	[161]
PLA/Hypericum perforatum oil (HPO)	Electrospinning	9% PLA was dissolved into a mixture of dichloromethane and acetone solvents of 50:50 (*v*/*v*); flow rate was 5 mL/h; voltage was 22 kV; distance between the needle tip and the collector was 15 cm; rotation of cylindrical drum was set at 35 rpm; ambient conditions: temperature was 25 ± 2 °C, and relative humidity was 50 ± 5%	>99.99 antimicrobial activity against *E. coli* and *S. aureus*	[21]
PLA/GO/Q electrospun scaffolds	Electrospinning	10% solution of PLA dissolved into a solvent mixture of DCM/DMF (1:9); voltage range was 25 to 26.6 kV; distance between the needle and collector was 12 cm; flow rate was in the range of 0.3 to 0.6 mL/h	Entire delivery of the loaded Q for 10 s of electric stimulation at 10 Hz and 50 Hz	[162]
PBSu/arabic, karaya, and tragacanth edible gum fibrous mats; PBSu/coriander and lavender essential oil fibrous mats; PBSu/linoleic acid fibrous mats	Electrospinning	Optimal conditions: Concentration of PBSu/DCH solution of 14% *w/w*; chloroform:methanol (90:10) solvent; voltage of 15 kV; flow rate of 1 mL h^−1^; distance between blunt stainless-steel needle and collector of 20 cm; ambient temperature of ∼20 °C and RH of 30%	All agents endowed polyesters mats with antimicrobial effects toward Gram-positive and Gram-negative bacteria and biocompatibility; all gums enhanced the mechanical properties to be similar to those of human skin	[15]
PBAT/PCL microfibers, loaded with 10 and 20% silver sulfadiazine (SS)	Electrospinning	75% PBAT/25% PCL were prepared in a mixture of solvents: chloroform (85% *v*/*v*) and DMF (15% *v*/*v*); flow rate was 1 mL/h; distance from needle to collector was 12 cm; positive voltage was 15 kV; ambient conditions: temperature was 23.5 ± 1.5 °C, and relative air humidity was 50 ± 5%	Antimicrobial assays against *E. coli* and *S. aureus*	[22]
PLCL/PCL/platelet lysate (PL) nanofiber membrane	Electrospinning	5% (*w*/*w*) PLCL and 5% (*w*/*w*) PCL in a mixture of chloroform/ethanol/acetic acid solution at an 8:1:1 ratio; voltages of −10 kV and +40 kV; distance between needle and collector was 190 mm; rewinding speed was 18 mm min^−1^; ambient conditions: temperature of 22 °C, and humidity of 50%	Enhanced the keratinocytes and endothelial cells	[163]
Poly(vinylpyrrolidone) (PVP)/PLA—poly(ethylene oxide) (PEO) dressing scaffold containing collagen and cefazolin as antimicrobial agents	Coaxial electrospinning	Core solution: 10% (*w*/*v*) mixture of PLA/PEO (80:20 *w/w*) was prepared in DCM/DMF (7:3, *v*/*v*), then cefazolin was added; Shell solution: 30% (*w*/*v*) PVP with different concentrations of collagen (10%, 20%, and 40% *w/w*) was prepared in HFIP/ethanol (7:3, *v*/*v*); flow rates for core and shell polymer solutions were 0.2 and 0.8 mL/h, respectively; voltage was 22 kV; distance from needle tip to collector was 15 cm	Antimicrobial activity against *E. coli*, *S. aureus*, and *Pseudomonas aeruginosa*	[16]
Polyester dressings (PD)/Annona leaf extracts (ALE) (2.6% and 5.3% concentrations of ALE to PD)	Supercritical solvent impregnation (SSI)	Impregnation: 5 or 10% ethanol concentration; temperature of 55 °C; pressure of 300 bar; CO_2_ flow of 10 g/min; time of 1 h Drying step: CO_2_ flow of 5 g/min for 30 min Fast depressurization of the cell: rate of 100 bar/min	Impregnation yield in the range of 0.4–0.82 mg ALE/100 mg PD; antioxidant loading 11.11–16.36 µg AOX/mg dressing; antibacterial activity against *S. aureus* and *E. coli*	[164]
PLA/1% babassu oil membrane—electrospinning (ES) PLA/babassu oil—solvent casting (SC)	Electrospinning Solvent casting	14% (*w/v*) PLA in a mixture of chloroform/N, N-dimethylformamide (8:2) solvents; distance from needle to collector was of 10 cm; flow rate was 0.5 mL/h; applied voltage was 18.5 kV; ambient conditions: temperature of 25 °C and humidity of 55%	Higher water vapor transmission rate (WVTR), maintaining a humid environment above the wound, good cytotoxicity, stimulating the keratinocyte migration, and inhibition of *Pseudomonas aeruginosa* growth for membrane obtained by ES as compared with SC	[19]
PLA/up to 1% CuO and ZnO NPs/2% tranexamic acid (TXA)	Electrospinning	10, 14, and 18 *w/v* % PLA were prepared in chloroform and DCM (3:1 ratio); flow rate was 0.5 mL/h; voltage was 20 kV; distance from needle to collector was 10 cm; rotation rate of collector was 400 rpm	*Improved hydrophilicity;* antimicrobial effect against *E. coli* and *S. aureus*	[20]
PHB/PCL/melanin–TiO_2_ nanostructures	Electrospinning vs. coating	68 mg/mL of PHB and 52 mg/mL of PCL were dissolved into chloroform:ethanol solvents at ratio of 4:1 *v*/*v* for 3 h; 74 mg/mL of nanoparticles were added into this solution; Voltage was ~3 kV; flow rates were 1400 μL h^− 1^ and ~340 μL h^− 1^ for electrospun mats and coated mats, respectively	Coated melanin–TiO_2_/PCL/PHB mats were more hydrophilic, and showed a higher water uptake than PCL/PHB mats; poor cytotoxicity toward HaCat eukaryotic cells; Antimicrobial activity toward both Gram (+) and Gram (−) strains	[18]
PHB/30% gelatin/0.2% (*w*/*v*) SS	Electrospinning	Solutions of 4% *w*/*v* PHB or gelatin were dissolved in HFIP; electric potential was 12 kV/cm; distance from needle to collector was 15 cm; flow rate was of 0.8 mL/h	Antimicrobial activity against *S. aureus*, *P. aeruginosa,* and *E. coli*; Fast healing of wound from day 18	[24]

**Table 4:** Examples of polyester-based materials obtained by different techniques for vascular applications.

Composition	Method	Features/Remarks	Application	Ref.
PLA	3D printing—FDM	Biocompatible and biodegradable vascular graft; modification of the flow rate of PLA led to different pore sizes and porosities; slow degradation of PLA allowed mechanical support in vivo for cell growth	Vascular grafts	[188]
PLA/human aortic smooth muscle cells	3D printing/self-organizing cell sheet method	Replication of tunica media; 11.5× increase in uniaxial ultimate tensile strength (UTS) compared with tunica media layer of a common iliac artery; spontaneous contraction of muscle cells—functional capacity of engineered rings	Blood vessel repair	[189]
PCL/(organoselenium modified polyethyleneimine/heparin) (SePEI/Hep)	PCL grafts—electrospinning; SePEI and heparin—layer by layer	In situ nitric oxide (NO) generation; increased adhesion and proliferation of endothelial cells; inhibited the adhesion of smooth muscle cells	Small-diameter vascular grafts (<6 mm)	[190]
PCL monolayer and PCL and PEG bilayer scaffolds	Electrospinning/co-electrospinning	Appropriate mechanical properties for in vivo implantation; PEG increased the porosity of the scaffolds, which favored cell proliferation on the inner-layer surfaces of the scaffolds	Vein grafts	[191]
PCL/collagen type I multilayered scaffolds	Bidirectional electrospinning	Mechanical properties comparable to native blood vessels; PCL was loaded with 15% vancomycin/16% gentamycin for decreasing postoperative infection; hemocompatible blood–scaffold interface	Arteriovenous vascular grafts for hemodialysis	[192]
PCL functionalized with heparin and vascular endothelial growth factor (VEGF)	Electrospinning	Antithrombogenic properties; association of heparin and VEGF with PCL scaffolds favored the endothelial layer formation and regeneration of damaged vessels	Vascular tissue engineering	[193]
PLCL	Electrospinning	Porosity ~70%, (pore of 9.34 ± 0.19 μm, fiber diameters of 5.58 ± 0.10 μm); in vitro adhesion and proliferation of endothelial cells; 6 months in vivo—vessel regeneration; but due to rapid rate of degradation—loss of mechanical properties	Bypass for the rabbit carotid artery	[194]
PLCL functionalized with heparin and substance P (SP), a neuropeptide	Electrospinning	Heparin—thrombogenic responses suppression; P (SP)—to recruit host cells; histological analysis—formation of new tissue, deposition of collagen and elastin, and a large number of blood vessels	Cell-free small-diameter vascular grafts	[195]
PCL/resveratrol	Electrospinning	Resveratrol—sustained and controlled release; vascular regeneration by modulation of endothelial cells and M2 macrophages	Abdominal aorta	[196]
PCL–chitosan (CTS) nanofibers coated with PCL stands	Electrospinning and extrusion (3D bioprinting)	PCL increased the strength of the artificial vessels; CTS enhanced hydrophilicity, allowing cell adhesion and proliferation	Biotubular scaffolds for artificial vascular grafts	[197]
Heparin-releasing PLLA (wall), PCL (reinforcement)	Electrospinning and extrusion (3D printing)	Tubular scaffold with D: 5 mm, L: 6 cm; heparin stimulated stem cell differentiation; no thrombosis, inflammation, or structural failure	Aortic vascular reconstruction	[198]
Poly(propylene fumarate) (PPF)/fibrin scaffold	Digital light processing (DLP)—3D printing	Cylindrical scaffolds (6 mm height, 0.25 mm wall thickness, 3 mm outer diameter, pore size 0.35 mm); increasing preculture time led to spread of vascular networks; biomaterial with stable mechanical properties (ultimate tensile strength of 1.48 MPa, elastic modulus of 8.79 MPa, similar to native femoral artery and saphenous vein)	Vascularized neobone tissue	[199]
Woven polyester grafts with different coatings—collagen and gelatin	Graft patches (5 × 5 mm^2^ square) inoculated with bacterial strains	In vitro and in vivo tests—more biofilm formation on collagen-coated polyester vascular grafts compared with gelatin-coated grafts; bacterial adherence in vitro	Prosthetic thoracic vascular grafts	[200]
Silk fibroin (SF)-coated PET	PET vascular grafts (1.5 mm diameter)—double-Raschel knitting method; PET graft coated with SF—by immersion in a mixed aqueous solution (50:50 *w/w* % ratio of SF and glycerin as porogen)	SF(Glyc)-coated PET graft was rapidly degraded in vivo (in rats), and remodeling to self-tissues was promoted compared with the gelatin-coated PET graft	Small-diameter artificial vascular graft	[201]

**Table 5:** Design and performance of polyester-based formulations used in ophthalmology.

Composition	Method	Features	Application	Ref.
Intracameral PCL implants loaded with (DE-117) ocular hypotensive agent	PCL thin films by spin-casting; four layers of PCL films—stacked to reached a thickness of 224 μm; DE-117 powder placed between two stacked films, heat-sealing the edges	Zero-order release of DE-117 over 6 months (release rate of 0.5 μg/day); in vivo biocompatibility; effective distribution of released drug in relevant ocular tissues (cornea, aqueous and vitreous humor, iris–ciliary body)	Glaucoma treatment	[210]
PCL/timolol maleate and brimonidine tartrate codelivery implant	Spin-casting	PCL films of 20 mm thickness for brimonidine compartment and of 40 mm for timolol compartment; intraocular pressure (IOP)-lowering effects of the implant for 13 weeks in vivo (3.4 ± 1.6 mmHg); acceptable ocular tolerance	Glaucoma therapy	[209]
PEA or PLGA injectable microspheres; PEA loaded with dexametasone	Emulsion solvent evaporation; freeze-drying to obtain solid microspheres of PEA (10–20 µm) and PLGA (20 µm)	Müller glia cell activation was most pronounced in PLGA-injected eyes; viability of retinal cells was not affected; majority of microspheres were degraded (TEM)	Intravitreal drug delivery	[204]
Erythropoietin-loaded PLGA/PLA microspheres	Encapsulation by solid-in-oil-in-water (S/O/W) method	No apoptotic cells in the injected retinas; no increased glial fibrillary acidic protein expression; biocompatible and safe for intravitreal injection in rabbits	Posterior segment ocular diseases	[211]
PLGA—drug delivery carrier of Rho kinase (ROCK) inhibitor Y-27632	Emulsion	Cell proliferation of cultured corneal endothelial cells—promoted by sustained release of Y-27632 from PLGA microspheres (over 7–10 days in vitro)	Treatment of corneal endothelial disease	[212]
Cyclosporine A (CsA)-loaded (mPEGhexPLA) nanocarriers (ApidSOL)	Nanosized micelles formed spontaneously in water; mPEGhexPLA and CsA dissolved in acetone/sonication	No immediate toxicity after repeated topical application in mice; reduced T-cell count and proliferation, IL-2 secretion of cells from ipsilateral lymph nodes; local and systemic immunosuppression	Autoimmune uveoretinitis	[213]
Spironolactone (SPL) loaded methoxy-PEG–dihexyl-iodide-PLA (mPEG–dihexPLA) micelle	Dissolved in acetone/sonication; SPL:copolymer ratios: 1:20, 1:40, and 1:60	mPEG–dihexPLA increased aqueous solubility of SPL and enhanced drug bioavailability; 0.1% SPL micellar formulations—stable 12 month at 5 °C; improved the extent of re-epithelialization	Corneal wound healing	[214]
Nanomicelles (NMs) of amino-terminated PEG-block-poly(D,L)-lactic acid and hydroxypropyl methylcellulose (NH_2_–PEG-b-PLA/HPMC) loaded with FK506 (tacrolimus)	Solvent-evaporation-induced self-assembly in aqueous solution; (mean diameter of 101.4 ± 1.3 nm)	Good sustained release (up to 80% after 200 h) and cumulative penetration (280.16 ± 7.33 μg cm^−2^); significant increase in the in vitro permeation amount compared with 0.05% FK506 suspension drops; higher concentration and longer retention of FK506 in ocular tissue; NMs—good anti-graft-rejection reaction in rats	Intraocular drug delivery	[215]
mPEG–PCL micelles loaded with axitinib (tyrosine kinase inhibitor)	Emulsion evaporation	Increased drug solubility; good histocompatibility; low toxicity; easy penetration into the cornea against angiogenesis; excellent corneal transport performance of PEG–PCL	Corneal-neovascularization-related corneal diseases	[216]

## Data Availability

Not applicable.

## Abbreviations

PLA	poly(lactic acid)
PDLA	poly(D-lactide)
PLLA	poly(L-lactide)
PDLLA	poly(D,L-lactic acid)
PCL	poly(ε-caprolactone)
PGA	poly(glycolic acid)
PLCL	poly(L-lactide-co-Ɛ-caprolactone)
PLGA	poly(lactic-co-glycolic acid)
PETG	poly(ethylene terephthalate glycol)
PHA	poly(hydroxyalkanoates)
PHB	poly(hydroxybutyrate)
PHBV	poly(hydroxybutyrate-co-valerate)
PBAT	poly(butylene-adipate-terephthalate)
P(3HO-co-3HD)	poly(3-hydroxyoctanoate-co-3- hydroxydecanoate)
PHN	poly(3-hydroxynonanoate)
P(3HB)	poly-3-hydroxybutyrate
PHBV	poly(hydroxybutyrate-co-hydroxyvalerate)
PHOHD	poly(hydroxyoctanoate-co-hydroxydecanoate)
PU	polyurethane
TPU	thermoplastic polyurethane
PUR	polyester urethane
HA	hydroxyapatite
HNTs	halloysite nanotubes
PHEMA	poly-2-hydroxyethyl methacrylate
EMA	ethyl methacrylate
PEPA	poly(ethylene phosphoric acid)
PPE	personal protective equipment
PBS	phosphate-buffered saline
SnOct_2_	stannous 2-ethylhexanoate
PEG	polyethylene glycol
TCP	β-tricalcium phosphate
PICT	poly(1,4-cyclohexanedimethylene isosorbide terephthalate)
ABS	acrylonitrile butadiene styrene
CE	cellulose
EPDM	ethylene propylene diene monomer
NBR	nitrile-butadiene rubber
PBAN	polybutadiene acrylonitrile
SBR	styrene-butadiene rubber
mPEG-PLA	methoxy-poly(ethylene-glycol)-poly(D,L-lactide) copolymer
mPEGhexPLA	methoxy-poly(ethylene-glycol)-hexyl substituted poly(lactic acid)
mPEG–dihexPLA	methoxy-poly(ethylene glycol)–dihexyl-iodide-poly(lactic acid)
NH_2_–PEG-b-PLA/HPMC	amino-terminated poly(ethylene glycol)-block-poly(D,L)-lactic acid and hydroxypropyl methylcellulose
PPF	poly(propylene fumarate)
SPL	spironolactone
PP	polypropylene
PAN	poly(acrylonitrile)
PC	polycarbonate
PS	polystyrene
HDPE	high-density polyethylene
PE	polyethylene
PVDF	poly(vinylidene fluoride)
PMMA	poly(methyl methacrylate)
PEPA	poly(ethylene phosphoric acid)
PVP	poly(vinylpyrrolidone)
PEO	poly(ethylene oxide)
PEA	poly(ester amide)
PGS	poly(glycerol sebacate)
PBSu	poly(butylene succinate)
PVA	poly(vinyl alcohol)
PAA	poly(acrylic acid)
SS	silver sulfadiazine
TXA	tranexamic acid
NEO	neomycin
DEE	diethyl ether
SeA	sebacic acid
HV	hydroxyvalerate
DCH	diisocyanahexane
PL	platelet lysate
PTMC	poly(trimethylene carbonate)
SC-PLA	stereocomplex crystallite poly(lactide)
BCP	calcium phosphate
PGCit	poly(glycerol citrate)
PEGSU	urethane-based PEGylated poly(glycerol sebacate)
PGSLP	poly(glycerol-co-sebacic acid-co-l-lactic acid-co-polyethylene glycol)
PANI	poly(aniline)
SePEI	organoselenium modified polyethyleneimine
Hep	heparin
TIPS	thermally induced phase separation
DFO	deferoxamine
PRP	platelet-rich plasma
MSC	mesenchymal stem cells
PEI	poly(etherimide)
SL	soybean lecithin
BMP2	bone morphogenetic protein 2
MBGN	mesoporous bioactive glass nanoparticles
TCP	tricalcium phosphate
AOI	2-isocyanatoethyl acrylate
FFF	fused filament fabrication
3D	three-dimensional
FDM	fused deposition modeling
DLP	digital light processing
CAD	computer-aided design
DICOM	Digital Imaging and Communications in Medicine
CIN	cinnamaldehyde
BG	bioactive glass
ALP	alkaline phosphatase
OCN	osteocalcin
BC	bacterial cellulose
BOPSC	bioactive osteo-polyester scaffold
AgNPs	silver nanoparticles
GO	graphene oxide
COL	collagen
CuONPs	copper oxide nanoparticles
CMs	cardiomyocytes
SBF	simulated body fluid
FDA	U.S. Food and Drug Administration
PDL	periodontal ligament
PCAN	proanthocyanidins
AUPs	acrylate-endcapped urethane-based polymers
HFIP	hexafluoroisopropanol
HPO	hypericum perforatum oil
ALE	Annona leaf extracts
NaCMC	sodium carboxymethyl cellulose
PD	polyester dressings
SSI	supercritical solvent impregnation
WVTR	water vapor transmission rate
ES	electrospinning
SC	solvent casting
DCM	dichloromethane
DMF	dimethylformamide
HFIP	1,1,1,3,3,3-hexafluoro-2-propanol
NO	nitric oxide
Q	quercetin
PL	platelet lysate
QAS	quaternary ammonium salt
PCAN	proanthocyanidins
KPF-NCs	kaempferol nanocrystals
VEGF	vascular endothelial growth factor
LAI	long-acting injectable
NSAIDs	nonsteroidal anti-inflammatory drugs
DXI	dexibuprofen
